# Mapping of Dietary Interventions Beneficial in the Prevention of Secondary Health Conditions in Spinal Cord Injured Population: A Systematic Review

**DOI:** 10.1007/s12603-023-1937-6

**Published:** 2024-01-04

**Authors:** S. Stojic, I. Eriks-Hoogland, M. Gamba, E. Valido, B. Minder, A. Chatelan, L.G. Karagounis, M. Ballesteros, C. Díaz, M. Brach, J. Stoyanov, N. Diviani, S. Rubinelli, C. Perret, Marija Glisic

**Affiliations:** 1 Swiss Paraplegic Research, Guido A. Zäch Str. 4, 6207, Nottwil, Switzerland; 2 Faculty of Health Sciences and Medicine, University of Lucerne, Lucerne, Switzerland; 3 Institute of Social and Preventive Medicine, University of Bern, Bern, Switzerland; 4 Graduate School for Health Sciences, University of Bern, Bern, Switzerland; 5 Public Health and Primary Care Library, University Library of Bern, University of Bern, Bern, Switzerland; 6 Department of Nutrition and Dietetics, Geneva School of Health Sciences, HES-SO University of Applied Sciences and Arts Western Switzerland, Geneva, Switzerland; 7 Mary MacKillop Institute for Health Research (MMIHR), Australian Catholic University (ACU), Melbourne, Australia; 8 Centro de Investigación en Red de Epidemiología y Salud Pública (CIBERESP), Madrid, España

**Keywords:** Spinal cord injury, nutrition, cardiovascular diseases, gastrointestinal health, functioning, neurological recovery

## Abstract

**Objectives:**

Individuals with spinal cord injury are at risk of secondary health conditions (SHC) that develop as a consequence of autonomic dysfunction, prolonged oxidative stress and inflammation, and physical inactivity coupled with inadequate energy and nutritional intake. SHC can be debilitating and even life-threatening, and its prevention remains one of the major challenges in the continuum of medical care of aging SCI population. An unhealthy diet is a major driver of inflammation, oxidative stress, and unfavourable metabolic status and may be a practical preventive target to tackle increased SHC risk post-injury.

**Aims:**

To provide a catalogue of dietary interventions beneficial in prevention of SHC among individuals with SCI by conducting a systematic review of the literature on dietary interventions and dietary supplementation in promoting health and well-being after the injury. In addition, we aimed to provide a summary of observational studies exploring the association between habitual diet (macro- and micronutrients intake and dietary patterns) and health patterns following the injury.

**Method:**

This review was registered at PROSPERO (University of York) with registration number CRD42022373773. Four medical databases (EMBASE.com, MEDLINE [Ovid], Cochrane CENTRAL, and Web of Science Core Collection) and Google Scholar were searched from inception until 11th July 2022. Studies were included if they were clinical trials or observational studies conducted in adult individuals with SCI and provided information of interest. Based on strength of the study design and risk of bias assessment (using the NIH tool), we classified studies from Level 1 (most reliable studies) to Level 4 (least reliable studies).

**Results:**

Of 12,313 unique citations, 47 articles (based on 43 original studies) comprising 32 interventional (22 RCTs, 3 NRCT, and 7 pre-post studies) and 11 observational studies (2 cohort studies, 2 case-control, 1 post-intervention follow-up study, and 6 cross-sectional studies) were included in the present systematic review. Twenty studies (46.5%) were classified as Level 1 or 2, indicating high/moderate methodological quality. Based on those studies, dietary strategies including high protein diet, intermittent fasting, balanced diet in combination with physical conditioning and electrical stimulation, and dietary supplementation including alpha-lipoic acid, creatine, vitamin D, and cranberry-derived supplements and probiotics were mapped as the most promising in prevention of SHC among individuals with SCI.

**Conclusions:**

To develop timely and effective preventive strategies targeting major SHC (e.g., cardiometabolic diseases, urinary tract infections) in SCI, further research is warranted to confirm the effectiveness of dietary strategies/interventions identified through the current systematic review of the literature.

## Introduction

Spinal cord injury (SCI) causes loss of sensory and motor function below the level of injury, leading to lifelong disability and disturbing overall health and quality of life of the affected individuals (1, 2). Individuals experiencing disabilities are prone to «disability-related secondary health conditions» (SHC) that resemble the health issues experienced by aging individuals but occur earlier, have atypical presentation, and are indicative of accelerated aging (3). These SHC are attributed to physiological, cellular, and molecular changes resulting from the disruption of the central nervous system coupled with environmental hazards and poor health behaviour. Pain, bowel and bladder dysregulation, osteoporosis, obesity, and cardiovascular problems are the most commonly reported SHC following spinal cord trauma (2, 4). Although many SHC occur already in acute/subacute injury phase, the frequency and severity of problems tend to increase with time since injury (5). For instance, with prolonged life expectancy, cardiovascular diseases (CVD) became one of the major causes of death in individuals aging with SCI (3, 6, 7, 8, 9). Increased CVD risk is attributed to high prevalence of metabolic syndrome (MetS) post-injury (ranging from 25% to 87%) (10, 11). In particular, atrophy of metabolically active tissues coupled with a mismatch between reduced energy requirement (from an injury level-dependent activity limitation) leads to excess subcutaneous, visceral and muscular fat mass deposition (12, 13, 14, 15, 16, 17, 18, 19). A high ratio of body fat mass to fat-free mass in SCI plays a central role in the development of MetS since adipose tissue directly (and indirectly) affects glucose and lipid metabolism, neurohormonal changes, and inflammation. MetS was also associated with higher risk of developing pressure ulcers, lower urinary tract infections, and gut microbiota disbalance, which may further increase the risk of developing SHC (20, 21, 22). Since most risk factors for developing MetS are non-modifiable (e.g., injury characteristics, age, sex, genetic predisposition), targeting abdominal obesity via lifestyle changes may be the most promising approach to improve long-term health in SCI population and decrease their risk of SHC.

In the general population, adherence to a healthy lifestyle (healthy diet, physical activity, less stress, and healthy body weight) at mid-life was associated with longer life expectancy without major chronic diseases such as CVD, T2D, and cancer (23). Several studies in SCI have shown that exercise positively affects dyslipidemia and insulin resistance but may not be sufficient to decrease body fat (24, 25, 26, 27). In addition, due to physical (e.g., paralysis below injury level) and environmental barriers to exercise after the injury, diet modification may be a more practical target for disease prevention (28). Although robust evidence reports that most individuals with SCI may not follow appropriate dietary recommendations post injury (14, 15, 16, 17, 18), evidence on how habitual diet and micro- and macronutrient intake or specific dietary interventions affect metabolic and neurohumoral changes in SCI individuals remains inconsistent. Furthermore, although the importance of healthy diet adherence is acknowledged in dietary recommendations in the context of cardiometabolic diseases (CVD, MetS and diabetes) after SCI (28, 29), the health benefits of nutrition intervention go far beyond cardiometabolic disease risk improvement; they are also linked with improved immune system function, better bone and gastrointestinal health and mental well-being (30, 31, 32, 33, 34). Therefore, the main aim of the current systematic review is to map the most promising dietary strategies and dietary supplements in the prevention and maintenance of SHC (beyond its benefits on cardiometabolic disease prevention). In addition, we aim to provide a summary of the evidence exploring the role of habitual diet (macro- and micronutrients intake and dietary patterns) in health and well-being of SCI individuals.

## Methods

### Data Sources and Search Strategy

Guidelines on systematic reviews and meta-analyses in medical research were followed to conduct the current review (35, 36). Reporting was done following the Preferred Reporting Items for Systematic Reviews and Meta-Analysis (PRISMA) (37) guidelines. Study protocol was registered in PROSPERO (CRD42022373773). An experienced librarian created the search strategy which combined terms related to SCI and nutrition/diet and dietary interventions/supplements (Appendix I). Given the interest in including all health benefits, no restrictions based on health outcomes were applied. The search was performed using the following online databases: EMBASE. com, MEDLINE (via Ovid), Cochrane CENTRAL, and Web of Science Core Collection from inception until 11th July 2022 (date last searched). The first 200 results from the Google Scholar search engine were downloaded to verify the search strategy. No language restrictions were applied. A manual search of the reference lists of included articles, as well as the references included in relevant systematic reviews in the field, was performed to find additional eligible studies.

### Study Selection and Eligibility Criteria

Intervention studies were eligible for inclusion if they: (i) were conducted in adult individuals with SCI (≥ 18 years of age); (ii) explored the effect of dietary interventions (e.g., anti-inflammatory diet) or dietary supplements (e.g., vitamins, minerals, and such others.) on all types of SHC, functioning and well-being outcomes, and (iii) were designed as randomized, non-randomized clinical trials and pre-post intervention studies without a control group. All interventions, dietary intervention alone or in combination with lifestyle or behavioral modification, were acceptable for inclusion. In addition, any control group was considered eligible for inclusion (e.g., placebo, usual care, or other lifestyle intervention such as physical exercise). Observational studies were included if they: (i) were conducted in adult individuals with SCI (≥ 18 years of age); (ii) explored the association between habitual diet (e.g., micro- or macronutrient intake) and all types of SHC (e.g., cardiometabolic health, bone and muscle health, cognition and mood etc.), functioning and well-being and (iii) were designed as cohort studies, case-control studies, nested case-control studies, or cross-sectional studies. Animal and in-vitro studies, case reports/case series, letters to the editor, reviews, study protocols, commentaries, and conference abstracts were excluded.

### Full-Text Screening and Data Extraction

Two reviewers independently evaluated titles, abstracts, and full texts based on inclusion and exclusion criteria. In cases of disagreement, the decision was made by consensus or after consultation with a senior reviewer. Data extraction of the information relevant to research questions was performed in parallel. The following details were extracted from included studies: main author, publication year, study location, study design, intervention duration/study follow-up, exposure/intervention and control type, personal characteristics such as age, sex, body mass index (BMI), health status/comorbidities, duration and type of SCI and information on health outcomes.

### Methodological Quality Assessment of Individual Studies

We used the National Heart Lung and Blood Institute (NHLBI) Quality Assessment Tool (38, 39) to evaluate the methodological quality of controlled clinical trials (e.g., randomized, non-randomized, and pre-post studies) and observational studies. This tool focuses on the study's internal validity and includes items to evaluate potential flaws in study methods or implementation. In particular, the tool assesses potential sources of bias (e.g., patient selection), confounding, study power, the strength of causality in the association between interventions and outcomes, and other factors. To evaluate the study quality, reviewers went through a checklist and selected yes,” “no,” or “cannot determine/not reported/not applicable” in response to each item on the tool. For each item where “no” was checked, reviewers were instructed to consider the potential risk for bias that the flaw in the study design or implementation may introduce. “Cannot determine” and “not reported” were also noted as representing potential flaws. Two reviewers performed the study quality assessment independently (in parallel), and any dissent in quality evaluation was discussed with a third (senior) reviewer. We classified studies as Level 1 (highest quality) to 4 (poorest quality) based on the strength of the study design and quality assessment. For example, RCTs classified as high quality were considered Level 1 of evidence, while RCTs evaluated as moderate quality were classified as Level 2 of evidence. In contrast, regardless of the quality assessment, cross-sectional studies were evaluated as Level 4 of evidence (considering that their major flaw is the reverse causation bias). A detailed description of the process is provided in Supplemental table 1.

### Synthesis of Evidence

Due to substantial heterogeneity across study populations, study design, and assessed health outcomes we determined that a statistical meta-analysis would be inappropriate. Instead, to visually present the effect sizes across individual studies, we present individual trial results using STATA and provide a narrative synthesis. To provide an overview of the evidence regarding the general effects of dietary interventions, for each dietary pattern/dietary intervention or dietary supplement, an evidence summary was drafted depicting its role in health in SCI individuals and the methodological quality of individual studies. The evidence originating from more reliable interventional studies (Level 1 and 2 studies) is discussed in the main text, whereas, the evidence from studies of low reliability (Level 3 and 4) is presented for information only. In addition, we provided an overview on the association between habitual diet and health outcomes in a separate section.

## Results

### Study Characteristics

**Figure 1:**
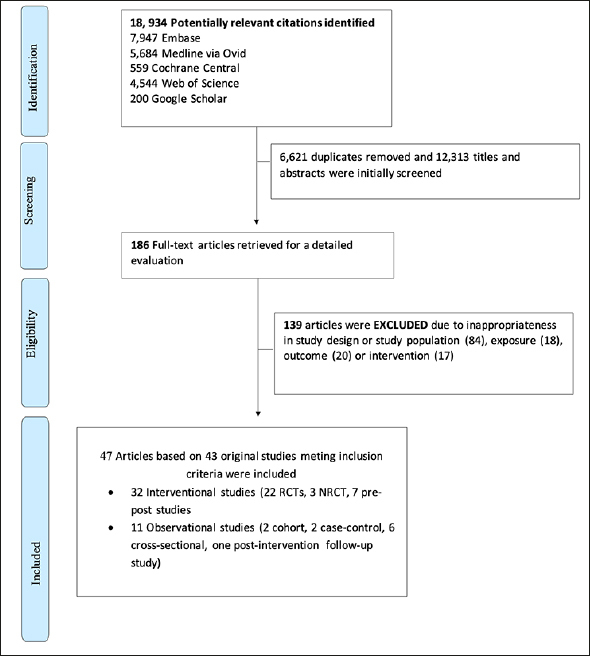
Flowchart of included studies

Of 12,313 unique citations identified through the search strategy, 186 relevant full-text articles were retrieved for further evaluation (Figure 1). Of these, 139 articles were excluded due to the following reasons: inappropriate study design or study population (n=84), inadequate exposure (n=18), or health outcome (n=20), or intervention (n=17). Thus, 47 articles based on 43 unique studies were included in the current systematic review. Among included studies, 32 were intervention studies (22 RCTs, 3 NRCT, and seven pre-post studies), and 11 were observational studies (two cohort studies, two case-control, one post-intervention follow-up study, and six cross-sectional studies).

**Figure 2:**
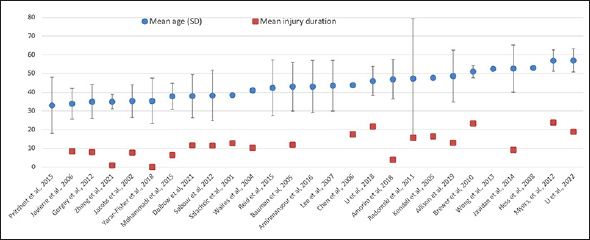
Mean age and injury duration accorss included intervention studies

Intervention studies characteristics are summarized in Table 1. Nineteen (59.3%) trials were conducted in North America, five in Iran, four in Europe, three in Australia, and one in China. Sixteen (50%) of studies had small sample size (≤30 participants), and intervention duration ranged from four weeks to 24 months. Clinical trials studied the effectiveness of (i) dietary strategies alone: low carb/high protein diet, anti-inflammatory diet, American Heart Association (AHA) diet, intermittent fasting; (ii) dietary strategies combined with physical exercise or nutrition counselling or (iii) dietary supplements: vitamin D, calcium, creatine, cranberry extract, omega-3 fatty acids, probiotics, and alpha-lipoic acid. Control groups varied across trials and overview can be found in Table 1. Among 32 clinical trials, the majority reported on cardiometabolic risk factors (n=16, 50%), urinary system (n=7, 21.8%), and musculoskeletal health and physical performance (n=9, 28.1%). We identified four trials on functional independence/neurologic recovery, and two on gastrointestinal health, depression, cognitive function and general well-being, and willingness to change, respectively. Twenty-three trials (71.9%) included individuals with cervical and thoracic injuries, seven included subjects with all injury levels, while two studies include individuals with cervical injuries. Sixteen trials (50%) included individuals with complete and incomplete injuries, six included motor complete injury only and ten studies did not report on injury completeness. Five trials included individuals with acute/subacute injury while the majority (n=27, 84.4%) focused on those with chronic injury. The mean time since injury was reported across 21 clinical trials and ranged from 0.9 years (SD 0.1 years) to 24 years (SD 12.3 years), Figure 2. Eight trials (25.0%) included only men, and the rest included predominantly male population. The mean age across 27 clinical trials ranged between 33 years (SD 15 years) and 57 years (SD 6.2 years), Figure 2. The most important findings are summarized in Supplemental table 2 & 3.

**Table 1:** General characteristics of intervention studies

**General characteristics**				**Personal characteristics**			**Injury characteristics**			**Experimental design**		
**Author, Year of Publication**	**Country**	**Study design**	**Population size (intervention, control)**	**Health status**	**Mean age ± SD**	**Number (%) of men**	**Injury level and completeness**	**Injury duration (years)**	**Intervention duration**	**Intervention type**	**Control type**	**Level of evidence**
Allison et al. 2019 (87) Allison et al. 2015 (65) Allison et al. 2017a (54) Allison et al. 2017b (41)	Canada	RCT	20 (12, 8)	Individuals experiencing unstable medical condition within 2 weeks before intervention were excluded	48.7 ± 13.9	10 (50%)	Cervical and thoracic, AIS A/B/C/D	13.10 ± 10.56	12 weeks	The anti-inflammatory diet which involved elimination of foods associated with common food intolerances and those that may increase inflammation. Daily supplements like Omega-3, Chlorella, Antioxidants, etc. were also provided	Habitual diet/no intervention	2
Aminmansour et al, 2016 (52)	Iran	RCT	64 (32, 32)	Involvement of the nerve roots, cauda equina only, gunshot wounds, life-threatening morbidity were excluded	43 ± 14	34 (53.1%)	Cervical, thoracic, lumbar, AIS NA	Acute injury	24 weeks	Intramuscular injection of progesterone 0.5 mg/kg twice a day for 5 days in addition to oral vitamin D (5 µg/kg twice a day for 5 days on admission) + Standard treatment with methylprednisolone (30 mg/kg intravenously as bolus dose and 15 mg/kg each 3 hours till 24 hours)	Placebo + Standard treatment with methylprednisolone (30 mg/kg intravenously as bolus dose and 15 mg/kg each 3 hours till 24 hours)	1
Amorim et al, 2018 (46)	Portugal	RCT, pilot	14 (10, 4)	NA	47 ± 10.6	13 (92.9%)	Cervical and thoracic, AIS NA	3.92 ± 0.87	12 weeks	Creatine (n=5), vitamin D (n=5) plus progressive resistance training with four different type of exercises aiming to strengthen the upper body	Placebo and progressive resistance training with four different type of exercises aiming to strengthen the upper body	2
Bauman et al, 2005 (51)	USA	RCT	40 (19, 20)	NA	43 ± 13	39 (97.5%)	Cervical, thoracic, AIS A/B	12 ± 10	24 months	1α-D21 (4 µg/day) (Bone Care International, Madison, WI). Calcium (1.3 g/d) and vitamin D (800 IU/d; 20 µg/d)	Placebo containing calcium (1.3 g/d) and vitamin D (800 IU/d; 20 µg/d)	2
Brewer et al, 2010 (88)	Australia	NRCT	35 (18, 17)	NA	51.1 ± 3.4	34 (97.1%)	Cervical and thoracic, AIS NA	23.32 ± 2.8	Until full wound healing occurred	Consumption the equivalent of two sachets of a commercially available arginine-containing powder (Arginaid, Nestlé Nutrition, Minneapolis, MN, US) per day. Each sachet of 9.2g containing 4.5g of arginine, 4g of carbohydrate, 155mg of Vitamin C and 60mg of vitamin E.	Historical control group (as assessed by medical history audit) – no intervention fruit, and vegetable) in order from high energy concentration	3
Chen et al., 2006 (89)	USA	Pre-post study	16 (16, no control)	Overweight or obese	43.8 (21.0–66.0)	9 (56%)	Cervical and thoracic, ASIA A/C/D	17.5 (1.7–60.3)	24 weeks	1200 kcal for women and 1400 for men within number of servings from five food groups (fat, meat/dairy, starch, to low energy concentration. 90-min education class once a week for 12 weeks. 30-min exercise session from week 6.	NA	4
Dolbow et al, 2021 (90)	USA	NRCT – pilot study	13 After drop out: 10 (5, 5)	NA	38 ± 11.5	3 (30%)	Cervical and thoracic, AIS NA	11.7 ± 7	8 weeks	RG-HIIT-FES cycling program (Resistance-guided (RG) HIIT2 into FES3 cycling) - 30 min 3 times per week and nutritional counselling - 30 min once per week (22.7 or 27.9 kcal/kg of body weight, 0.8–1.0 grams of protein per kilogram of body weight per day, and 1 milliliter of fluid per kilogram of body weight plus 500 milliliters per day).	Nutritional counselling (22.7 or 27.9 kcal/kg of body weight, 0.8–1.0 grams of protein per kilogram of body weight per day, and 1 milliliter of fluid per kilogram of body weight plus 500 milliliters per day).	3
Gorgey et al., 2012 (42)	USA	RCT, parallel	9 (5, 4)	Otherwise healthy	35 ± 9	9 (100%)	Cervical and thoracic, AIS A/B	8 ± 10	12 weeks	Resistance Training (leg extensions using surface Neuromuscular electrical stimulation (NMES) and ankle weights) and diet (45% carbohydrate, 30% fat, and 25% protein)	Diet alone (45% carbohydrate, 30% fat, and 25% protein)	2
Hess et al., 2008 (59)	USA	RCT, crossover	57 started 47 completed study	NA	53 years (range: 28–79)	47 (100%)	Cervical and thoracic, AIS A/B/C	Chornic injury, (mean value NA)	24 weeks (crossover without washout time)	Cranberry extract tablet (500mg) twice per day	Placebo	2
Jacobs et al., 2002 (49)	USA	RCT, crossover	16 (8, 8)	NA	35.3 ± 8.6	16 (100%)	Cervical, AIS A/B	7.75 ± 6.5	7 days (a 21-day washout period in between)	20g/d of creatine monohydrate supplement powder (1 teaspoon of the respective supplement with 8 ounces of water, 4 times daily)	Placebo	2
Javidan et al., 2014 (53)	Iran	RCT	104 (54, 50)	No history of diabetes, cancer, endocrinology disease, acute infection, etc.	52.7 ± 12.6	85 (81.7%)	Cervical, thoracic, lumbar, AIS NA	9.2 ± 6.3	14 months	MorDHA capsules (435 mg of docosahexaenoic acid and 65 mg of eicosapentaenoic acid) per day. No specific advices on food intake were given to patients and no diet modification was made through the study.	Two placebo capsules - twice daily No specific advices on food intake were given to patients and no diet modification was made by us through the study.	1
Javierre et al., 2005 (91)	Spain	Pre-post study	19 (19, no control)	NA	NA	19 (100%)	Cervical and thoracic, ASIA A/B/C/D	Chronic, (mean value NA)	24 weeks	Oral supplementation with the DHA (1.5 g) + EPA (0.75g) mixture given 6x day (2 per each meal)	NA	4
Javierre et al., 2006 (92)	Spain	Pre-post study	21 (21, no control)	NA	33.9 ± 8.2	21 (100%)	Cervical and Thoracic, AIS NA	8.5 (4.8 to 25.0 Years)	6 months	Daily supplement of 1.5 g·day-1 of docosahexanoic acid (DHA) and 0.60 g·day-1 of eicosapentaenoic acid (EPA) plus 9 mg of α-tocopherol	Evaluation procedure: control 1 - starting point; control 2 – at 3 months; control 3 at 6 months.	4
Kendall et al., 2005 (50)	USA	RCT, crossover	8 (4, 4)	No cognitive deficits and history of diabetes mellitus or renal dysfunction	47.8	7 (87.5%)	Cervical, AIS A/B/B	16.5	7 days (a 5-week washout period in between)	20g (10 g 2x/day) of creatine monohydrate powder (America's Nutrition, Rochester Hills, Ml) for 6 days, then maintained on 5 g daily	Placebo	2
Lee et al., 2007 (55)	Australia	RCT	305 (75, 78, 75, 77)	Subjects with neurogenic bladder and stable bladder management	43.5 ± 13.5	252 (83%)	Cervical and thoracic, AIS A/B/C	Median time since injury: 12 years (range 1 month to 61 years)	24 weeks	The urinary antiseptic Methenamine Hippurate (MH) 1 g twice-daily. Cranberry tablets 800 mg twice-daily. Factorial design: Group 1: MH with Cranberry. Group 2: MH with Cranberry. Placebo Group 3: Cranberry with MH placebo.	Group 4: MH placebo with Cranberry placebo	1
Li et al., 2018 (40)	USA	RCT, pilot	11 (5, 6)	Included individua with type 2 diabetes (n=3, 27.3%)	46.0 ± 7.8	10 (90.9%)	Cervical and thoracic, AIS A/B	21.8 ± 6.3	8 wk	A 8-week iso-caloric high-protein diet: ∼30% total energy as protein (1.6 g/kg per day)	Combined exercise regimen - 3 days/week	3
Li et al., 2022 (62)	USA	RCT (preliminary results)	25 (12, 13) Analysed: 19 (8, 11)	Participants had impaired glucose tolerance or insulin resistance, had no type 2 diabetes and no kidney disease	56.96 (6.2)	13 (68.42%)	Cervical and thoracic, AIS A/B/C	19.04 (13.1)	8 weeks	Low-carbohydrate, high-protein (LC/HP) diet that includes healthy dietary components (e.g., lean meat, whole grains, fruits and vegetables, fiber, etc.)	Not receive any dietary intervention (continue with their regular diet)	2
Linsenmeyer, et al, 2004 (58)	USA	RCT, crossover	21	Individuals with neurogenic bladders due to SCI.	NA	16 (76.2%)	Cervical, thoracic, lumbar, AIS NA	Chronic injury, (mean value NA)	4 weeks (1 week washout period in between)	1200 mg cranberry tablet (400-mg cranberry tablet 3 times/day)	Placebo	2
Mohammadi et al., 2015 (45)	Iran	RCT	58 (28, 30)	No self-reported specific diseases and malignancies	37.9 ± 7.0	58 (100%)	Cervical and thoracic, AIS A/B	6.4 ± 2.8	12 weeks	600 mg of alpha-lipoic acid (ALA) supplementation	Placebo	1
Myers, et al., 2012 (93)	USA	Pre-post study	26	Relatively healthy	56.92 ± 5.74	26 (100%)	Cervical and thoracic, ASIA A/B/C/D	23.8 ± 12.3	24 months	After recruitment and initial testing, participants underwent a baseline visit that included blood analyses; dietary, lifestyle, and physical activity questionnaires; a maximal exercise test; an evaluation by a physical therapist; and recommendations for individualized exercise and nutrition plans a physical therapist; and recommendations for individualized exercise and nutrition plans	NA	4
Pritchett et al., 2015 (94)	USA	Pre-post study	34	Para-athletes	33 ± 15	NA, male and female	Cervical, thoracic, lumbar, AIS NA	Chronic injury, (mean value NA)	12 and 16 weeks	Participants with deficient 25(OH)D status (<50 nmol/L) received 50,000 IU/week for 8 weeks, and participants with insufficient status (50–75 nmol/L) received 35,000 IU/week for 4 weeks, after which both received a maintenance dose of 15,000 IU/week. Participants with sufficient status (>75 nmol/L) received the maintenance dose of 15,000 IU/week. 25(OH)D concentrations increased significantly (p<.001; 66.3 ± 24.3 nmol/Land 111.3 ± 30.8 nmol/L pre- and post-supplementation, respectively	NA	4
Radomski et al., 2011 (64)	USA	Pre-post study	10 (10, no control)	NA	47.3 ± 32	6 (60%)	Thoracic, lumbar, ASIA A/B	15.7 ± 29.04	12 weeks (end of program). 24 weeks (long-term follow-up).	Each week, participants attended a class, group exercise session, and an individual exercise session The nutrition component emphasizes development of and adherence to an individualized meal plan that meets nutritional, medical, and weight management goals.	NA	4
Reid et al., 2015 (95)	Canada	Pre-post study	15 (15, no control)	NA	42.3 ± 14.9	10 (66.7%)	Cervical, thoracic, AIS NA	Chronic injury, (mean value NA)	15 days	250 ml glass of water in addition to their normal diet, at breakfast, lunchtime and dinner time for 7 days. On the ninth day, for a further 7 days, each patient took a 250-ml glass of cranberry juice (Ocean Spray Cranberries, Lakeville, MA, USA) at the three meal times.	NA	4
Sabour et al, 2012 (47)	Iran	RCT	75 (39, 36)	Individuals with osteoporosis	38.28 ± 13.52	69 (84,14%)	NA	11.55 ± 19.44	4 months	Two MorDHA capsules (435 g of DHA and 65 mg of EPA) per day plus 1000 mg calcium and 400 IU vitamin D daily.	Placebo plus 1000 mg calcium and 400 IU vitamin D daily.	1
Sabour et al., 2018 (44)	Iran	RCT	57 (30, 27)	Individuals with body mass index higher than 22 kg/m^2^	NA	45 (78.9%)	Cervical, thoracic, lumbar, AIS NA	Chornic injury, (mean value NA)	7 months	Standard nutrition brochures and education program (5 sessions). Specific diets designed for each individual based on anthropometric characteristics	Standard nutrition brochures	1
Sappal et al., 2018 (57)	USA	RCT	13 (7, 6)	Subjects without urolithiasis. Also excluded if they had fevers, chills, nausea, vomiting, anorexia, generalized malaise, new or worsened spasticity, autonomic dysreflexia, or subjective sense of having a UTI	> 65 (mean age NA)	16 (100%)	Cervical and thoracic, AIS A/B/B	> 6 months post injury (mean value NA)	15 days	Daily dosage of concentrated proanthocyanidins (PACs) in the cranberry supplement ellura	Placebo	2
Szlachcic et al., 2001 (29)	USA	NRCT	222 (86, 136)	NA	38.5 ± 11.1	198 (89.2%)	Cervical and thoracic, AIS A/B/C/D	12.8 ± 8.3	96 weeks	Diet based on recommendations of the American Heart Association (AHA) and American Dietetic Association guidelines.	No dietary consultation	4
Toh et al., 2019 (60 61)	Australia	RCT	207 Group 1: n = 51 Group 2: n = 50 Group 3: n = 53 Group 4: n = 53	Individuals with stable neurogenic bladder management	49.1	164 (79%)	Cervical, thoracic, lumbar, AIS A/B/C/D	Chornic injury, (mean value NA)	24 weeks	Group 1: Lactobacillus reuteri RC-14+Lactobacillus GR-1 (RC14-GR1), concentration per capsule was 5.4 × 109 colony-forming units + Lactobacillus rhamnosus GG+Bifidobacterium BB-12 (LGG-BB12), concentration per capsule is 7 × 109 colony-forming units Group 2: RC14-GR1 (concentration as above) + matched placebo (no LGG-BB12) Group 3: LGG-BB12 (concentration as above) + matched placebo (no RC14-GR1)	Group 4: Matched placebo capsules	1
Waites et al., 2004 (56)	USA	RCT	26 (10, 16)	NA	41 (NA)	42 (91.3%)	Cervical, thoracic, AIS A/B/C/D	10 (NA)	24 weeks	2 g of concentrated cranberry extract in capsules (divided into 2 dailly doses)	Placebo	2
Wong et al., 2013 (63)	UK	RCT	158 (78, 82)	Individuals with diarrhoea before antibiotic therapy, antibiotics or probiotic for prophylaxis, bowel problems, infective endocarditis and immunosuppression were excluded	52.5	131 (82.9%)	Cervical, thoracic, lumbar, AIS A/B/C/D	Sustained injury less than 6 months prior to trail	While using antibiotics +7 days	Probiotic drink (Yakult Light®: 65 ml) containing a minimum of 6·5 × 109 colony-forming units (CFU) LcS/bottle and skimmed milk, for the duration of the antibiotic course. In addition, probiotic was used for additional 7 days	Routine care for duration of antibiotic course	2
Yarar-Fisher et al., 2018 (48)	USA	RCT, pilot, feasibility	7 (4, 3)	NA	35.4 ± 12.2	5 (71.4%)	Cervical and thoracic, ASIA A/B/C	16 days ± 7.9	5 weeks	Ketogenic diet (KD): a high-fat, low-carbohydrate diet (≈72% total energy as fat, ≈25% as protein, and ≈3% as carbohydrate during enteral feeding and ≈65% total energy as fat, ≈27% as protein, and ≈8% as carbohydrate and fiber during solid feeding	Standard diet (SD): ≈35% total energy as fat, ≈27% as protein, and ≈44% as carbohydrate and fiber	2
Zheng et al., 2021 (43)	China	RCT	37 (19, 18) Completed: 34 (16, 18)	Individuals with respiratory failures and diabetes were excluded	35 ± 3.9	34 (91.89%)	Cervical and thoracic, AIS A/B/C	0.9 (0.1)	8 weeks	Every-other-day fasting: Fasting lasted from 09:00 P.M. on day 1 to 06:00 P.M. on the following day (day 2). On day 2, breakfast and lunch were skipped and dinner was restricted to 30% of the daily average calorie intake	No food restrictions	2

Characteristics of eleven observational studies and the most important findings are summarized in Table 2. Most studies were conducted in North America (n=8, 72.7%), two in Iran and one in the Netherlands. Sample size varied from 20 to 2,976 SCI individuals, and most studies reported on the association between habitual diet and CVD risk factors (n=9, 81.8%). The mean age varied between 36 years (SD 10 years) and 53.3 years (SD 5.7 years) and all studies included individuals with chronic SCI.

**Table 2:** The general characteristics of observational studies

**Author, Year of Publication**	**Country**	**Study design**	**Population size**	**Health status**	**Mean age ± SD**	**Number (percentage) of men**	**Injury type, level and completeness**	**Injury duration (years)**	**Exposure**	**Statis-tical analysis**	**Adjustment**	**The main findings**	**Level of evidence**
DiPiro et al, 2019 (69)	USA	Cohort	2,979	Not described	NR	NR	Cervical and non-cervical, AIS not reported	12.1 ± 9.4	Six behavioral domains (prescription medication usage, alcohol use, smoking, two nutrition factors, and fitness)	Cox regression	No	- Study explored the association between two nutrition factors and CVD mortality. Nutrition — 1 factor (mostly healthy nutrition practices) reflected drinking juices, eating fruit, salad, carrots, vegetables, and breakfast; nutrition — 2 factor (mostly unhealthy options) reflected eating fried food, red meat, junk food, and adding salt to food. The hazard of death due to heart and blood vessel diseases was lower among those who reported higher scores on the nutrition — 2 factor, which primarily reflects unhealthy eating behaviours like eating fried, fast food, or red meat and adding salt to food.	3
de Groot et al., 2013 (67)	The Netherlands	Cohort	130	Without progressive disease	40.1 ± 13.8	70%	Cervical and thoracic, AIS A/B/C/D	<1 year since injury	Fat intake	Regression	No	- Self-care question related to fat intake at 1 year after inpatient rehabilitation discharge, was not associated with lipid levels nor body mass index.	3
Beal et al. 2017 (74)	USA	Case-control	20	Not described	47 ± 10.1	20 (100%)	Cervical, thoracic and lumbar, AIS A/B	15.2 ± 11.8	Vitamin D intake	Correlation	Total dietary intake, body weight	- Total cholesterol was lower in the higher vitamin D intake group as compared to lower intake group (148± 14.12 mg/dl vs. 171.8± 36.22). No differences were observed among other lipids. - Vitamin D intake adjusted to total dietary intake was positively correlated to Si adjusted to body weight (r= 0.63), Si adjusted to lean mass (r= 0.57), Sg (r= 0.53) and Sg adjusted to body weight (r= 0.52).	3
Lieber-man et al., 2014(70)	USA	Case-control	100	NA	45.3 ± 5.1	78 (78%)	Cervical and thoracic, AIS A/B/C	15.1 ± 9.6	Selected food group intake and CVD risk factors in SCI vs. age-, sex-, and race-matched ABI	Regression	Age, sex, race, center, education, energy intake, and physical activity	- There were not any statistically significant relations between individual cardiovascular risk factors (body anthropometrics, blood pressure, blood lipids, glucose, insulin and high sensitivity c-reactive protein) and food groups (vegetables and fruits, while grains, dairy and meat)	3
Gorgey et al., 2015 (71)	USA	Cross-sectional	16	Not described	38 ± 9	16 (100%)	Cervical and thoracic, AIS A/B	NR	Average caloric intake (in kcal) and percentage of macronutrients (carbohydrates, fat, protein)	Regression	No	- Total caloric intake was not related to any of the body composition variables or basal metabolic rate. The percentage of dietary fat was negatively related to the percentage of whole-body lean mass and percentage of trunk lean mass. The percentage of carbohydrates was negatively related to the percentage of whole-body fat mass, percentage of leg fat mass and percentage of trunk fat mass.	4
Abilmona et al., 201873	USA	Cross-sectional	22	Not described	36± 10	22 (100%)	Cervical and thoracic, AIS A/B	NR	Total caloric intake, total macronutrients intake (carbohydrate, fat and protein intake) and caloric surplus	Correla-tion	Antero-lateral trunk muscle ratio	- Total fat, carbohydrate and protein intake were negatively correlated with visceral adipose tissue (VAT) (Pearson r were −0.49, −0.51 and −0.63, respectively) - Total carbohydrate itake was negatively correlated with subcutaneous adipose tissue, SAT (Pearson r was −0.47) - No significant correlation between total fat and protein intake and fasting insulin were observed. After adjustement for trunk muscle ratio (TMR), total fat and total protein intake were positively correlated with fasting insulin (r=0.47 and 0.61, respectively) - Total caloric intake (r=−0.54 and −0.41) and surplus were negatively correlated with was negatively corelated with VAT and SAT(r=−0.43 and −0.44) respectively	4
Javidan et al., 2017 (72)	Iran	Cross-sectional	265	Without chronic medical conditions (e.g. diabetes, cancer, endocrinology disease, acute infection and etc.)	36.25 ± 10.76	217 (81.9%)	Cervical and thoracic, AIS not reported	NR	Protein intake	Correlation	No	- Higher carbohydrate, cholesterol and fat intake were associated with higher blood pressure. Higher carbohydrate intake was correlated with higher triglyceride levels and higher fat intake was correlated with higher LDL cholesterol. - Some amino acids had positive some had negative association with cardiometabolic risk factors. Eg., Higher intakes of threonine and leucine had a negative relationship with TG level; while lysine was positively related to levels of fasting plasma glucose, triglyceride, systolic and diastolic blood pressure	4
Li et al., 2021 (68)	USA	Cross-sectional	24	Without type 2 diabetes and active pressure ulcers	45 ± 12	16 (66.7%)	Cervical and non-cervical, AIS not report-ed	20 ± 13	High-protein/low-carbohydrate diet	Regression	Sex, level of injury, and body fat percentage	- Each 10-point increase of the Healthy Eating Index (HEI-2015) was associated with a 3.3-mg/dL decrease in fasting glucose concentrations. - No significant associations were observed between HEI-2015 and other cardiovascular risk factors	4
Goldsmith et al., 2022 (96)	USA	Cross-sectional study (baseline assessment of individuals involved in clinical trials)	48	Cardio-vascular disease, uncontrolled type 2 diabetes (or requiring insulin), haematocrit > 50 % or symptoms of a urinary tract infection were excluded	38 ± 12	42 (87.5%)	Cervical and thoracic, AIS A/B/C/D	10 ± 10	The mean macronutrient intake including fats, proteins and carbohydrates	Regression	No	- Lean mass (LM) measures were not significantly associated with any macronutrient combination. - Carbohydrates showed significant associations with percentage of total dat mass (%total-FM), total-FM, %trunk-FM, trunk-FM, percentage of lower extremity fat mass percentage (%LE-FM), and LE-FM - Interactions were found between carbohydrate x fat for LE-FM and protein × carbohydrate for %LE-FM and LE-FM - Linear regressions adjusted for total energetic intake did not influence the relationship between carbohydrate intake and measures of fat mass.	4
Allison et al., 2018 (66)	Canada	Post-intervention follow-up study	5	Not described	50.6 ± 11.8	4 (80%)	Cervical and thoracic, AIS A/B/C/D	12.8 ± 11.3	Anti-inflammatory diet compliance	ANOVA, descriptive statistics	No	- There was a significant reduction in diet compliance at the 1-year follow-up in comparison to the end of the dietary intervention at 3 months (92.6% versus 43.0%, p < 0.01). - Center for Epidemiological Studies Depression Scale (CES-D) scores showed a trend towards an increase from 3 months to follow-up (8.0 vs. 21.4, p = .10), with follow-up scores no longer statistically different from baseline (p = 0.74). - Sensory Neuropathic PainQuestionnaire (NPQ) scores remained unchanged from 3 months to follow-up (25.2 vs. 29.1, p = 0.42) but were still significantly lower than baseline (p = 0.02). Affective NPQ scores significantly in-creased from 3 months to follow-up (27.7 vs. 40.1, p = 0.05). Sensitivity NPQ scores showed no significant change from 3 months to follow-up (28.2 vs. 33.5, p = 0.34) but returned to a score similar to baseline (p = 0.15)	4
Mohammadi et al., 2021 (75)	Iran	Cross-sectional	150	Without amputation and specific chronic diseases and malignancies	53.3 ± 5.7	150 (100%)	Cervical and thoracic, AIS A/B/C/D	NR	Common portion sizes and the average reported frequency were used to determine the amount of food consumed.	t test and Chi-square tests, linear and logistic	Age, body mass index and energy, SCI level, completeness, smoking, education, marital statusand supplement use	- A one-unit increase in the dietary inflammatory index (DII) was significantly associated with higher scores of depressive symptoms (β = 1.31, 95% CI 0.44–2.18; P = 0.003). - A one-unit increase in the DII was associated with higher odds of having at least mild depressive symptoms (OR= 1.77, 95% CI 1.17, 2.67; P= 0.007). - No significant association was reported between the DII and anxiety and stress.	4

### Critical appraisal of methodological quality of included studies

Based on the NIH assessment, the most of clinical trials were classified as of moderate methodological quality (n=24, 75%), six trials were classified as of high quality and two as low. Two major issues in study quality comprised lack of power calculation and lack of study personnel blinding (Supplemental table 4). Most observational studies were classified as moderate methodological quality (n=6, 54.5%), and five were classified as low quality (Supplemental Table 5). Major issues referred to study participants selection (e.g., not transparent or convenience sampling used) and lack of adjustment for potential confounders. Based on strength of the study design and risk of bias assessment, we classified studies as Levels 1 to 4 (details on classification are provided in Supplemental Table 1). Among intervention studies 6 (18.6%) and 15 (46.9%) were categorized as Level 1 and Level 2, respectively; three were categorized as Level 3, and eight were categorized as Level 4. Amid observational studies, most were classified as Level 4 (n=7, 63.6%), whereas four were classified as Level 3.

### Cardiometabolic Risk Factors

**Figure 3:**
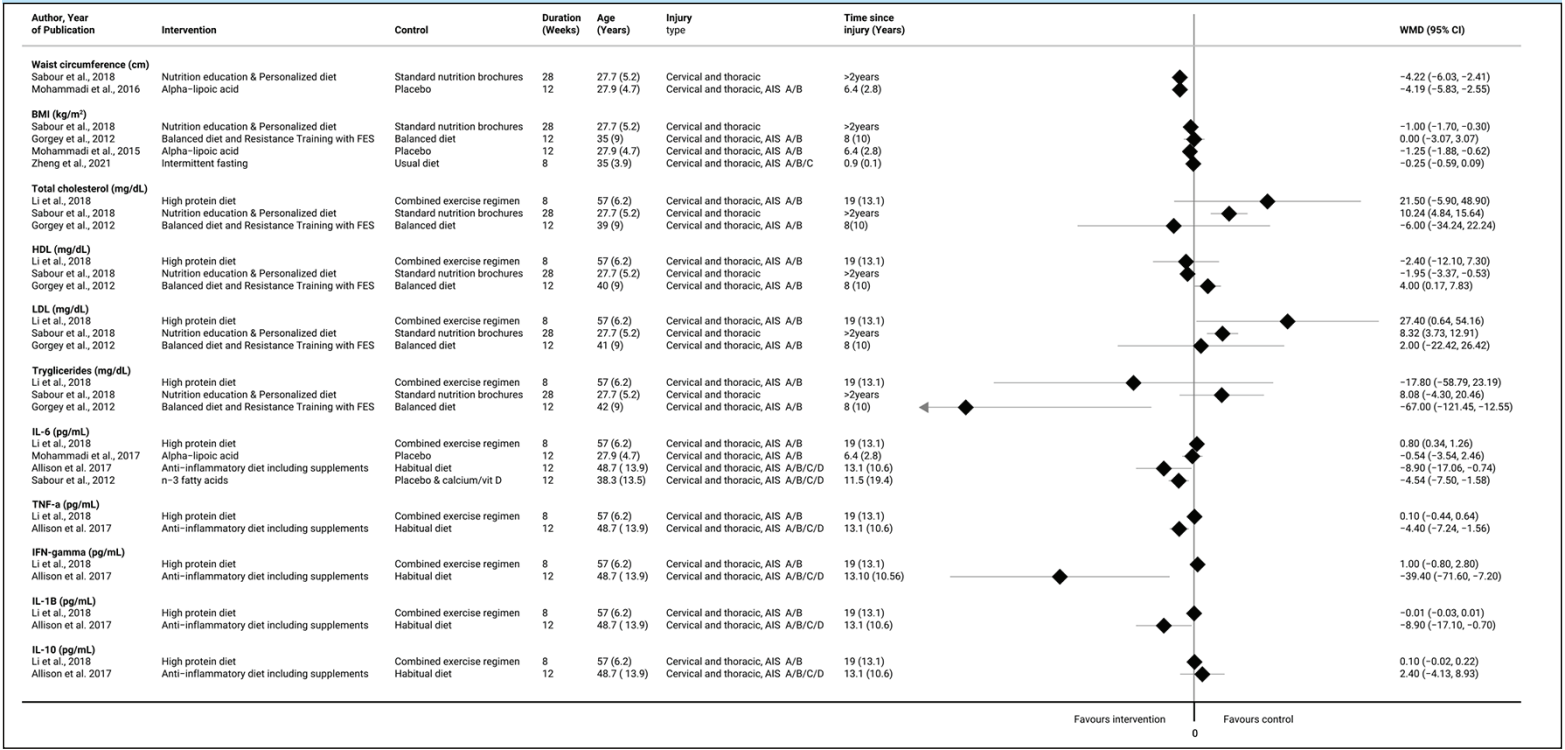
Changes in cardiovascular risk factors at each RCT's longest follow-up (without meta-analysis)

**Figure 4a and b:**
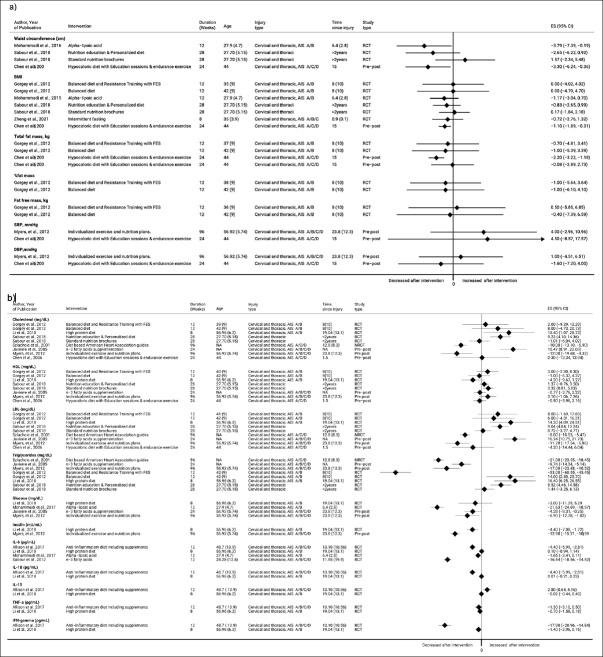
Mean difference in cardiovascular risk factors at the end of each intervention longest follow-up

Twenty-five studies, including 16 clinical trials (nine RCTs, three NRCT, and four pre-post studies) and 9 observational studies (6 cross-sectional, one case-control, and two cohort studies) reported on cardiometabolic risk factors. Most intervention studies were classified as Level 1 & 2 (n=8, 50%), whereas all observational studies were classified as Level 3 & 4. The evidence summary from Level 3 & 4 intervention studies can be found in Supplemental table 2. The Figure 3 and Figure 4 a & b provide the overview of mean changes in CVD risk factors across intervention studies. Herewith, we focus on evidence originating from Level 1 and 2 studies.

The forest plots depict mean difference in outcomes comparing beginning and end of intervention period (of intervention arm of RCTs or of pre-post studies) considering the longest follow up. Only outcomes with ≥2 studies were considered in analysis. Negative values indicate decrease in outcome at the end of intervention period and vice versa.

The forest plot depicts mean difference in outcomes between intervention and control group considering the longest follow up. Only outcomes with ≥2 studies were considered in analysis. Negative values indicate higher decrease in intervention group as compared to control group and vice versa.

In a small pilot study among eleven individuals with chronic motor complete injury, an 8-week high protein iso-caloric diet (∼30% total energy as protein) improved insulin sensitivity and decrease circulating levels of pro-inflammatory cytokine tumour necrosis factor-alpha (TNF-a) and total fat mass in SCI individuals with T2D (40). Similarly, a 12-week anti-inflammatory diet (including elimination of inflammation-inducing foods and foods and supplements with established anti-inflammatory properties) led to significant reduction of pro-inflammation markers (interleukin 6, IL-6, IL-1B, interferon gamma, INF-gamma) as compared to the control group (habitual diet). In addition, no significant differences in anti-inflammatory markers were observed between the two groups (41). An RCT compared changes in cardiometabolic risk factors after a 12-week intervention with balanced diet and balanced diet in combination with physical conditioning and neuromuscular electrical stimulation. In individuals who followed balanced diet without excercise, a significant improvement in response to a glucose load was observed (decrease in glucose area under the ROC curve after oral glucose tolerance test), and nosignificant changes were observed in fasting glucose, lipids, and body composition. The group receiving both a balanced diet and physical conditioning had a significantly higher decrease in Cholesterol/HDL ratio, triglycerides, and glycaemic response compared to the balanced diet intervention. In addition, a significant improvement in regional fat-free mass and a decrease in regional fat mass were observed in this group (42). In another RCT, an 8-week intervention with intermittent fasting led to a significant decrease in fasting glucose and body weight but did not influence BMI (43). A 7-month nutritional education with personalized diet in otherwise healthy SCI individuals did not influence blood pressure, lipid profile or anthropometric measurements (44). A clinical trial comparing a 12-week intervention with 600 mg of alpha-lipoic acid (ALA) supplementation and placebo reported a significant decrease in fasting glucose, waist circumference, BMI, and blood pressure in the intervention arm as compared to the control group; no differences were observed in high sensitivity c-reactive protein (hs-CRP) and IL-6 between the groups (45). Another RCT supplementing creatine (3 g/day) and vitamin D (25000 IU every two weeks) for eight weeks, accompanied by progressive resistance training, led to decreased skinfold thickness (46). A study exploring the role of n-3 polyunsaturated fatty acids with calcium and vitamin D in modifying pro-inflammatory cytokines in individuals with SCI and osteoporosis reported no significant changes after an 8-week intervention (47). Finally, a pilot study aiming to test the safety and feasibility of a ketogenic diet intervention (≈70–80% total energy as fat) in the acute stages of SCI, reported improvement in glucose levels;, additionally individuals in the intervention group managed to maintain normal levels of blood lipids whereas those in the control group experienced worsening of lipid profile (48).

### Musculoskeletal Health, Physical Performance, and Cardiorespiratory fitness

We identified eight interventional studies (4 RCTs, one NRCT, and three pre-post studies), of which four were Level 2 and four were Level 4 studies, exploring the effectiveness of dietary interventions/supplements on musculoskeletal health, physical performance and cardiorespiratory fitness (Supplemental Table 2). A trial supplementing creatine (3 g/day) and vitamin D (25000 IU every two weeks) for eight weeks accompanied with progressive resistance training improved arm muscle area and physical performance measured via the seated medicine ball throw, one-repetition maximum test for chest press, triceps, pec deck, and lat pulldown (46). No changes in study outcomes were observed in the control arm (progressive resistance training and placebo) (3). Similarly, short-term creatine monohydrate supplementation enhanced the exercise capacity in persons with complete cervical SCI measured through oxygen uptake (V·O2), carbon dioxide production (V·CO2), tidal volume (VT), and ventilatory frequency (49). In another cross-over trial in individuals with tetraplegia and mild wrist extensor weakness, creatine supplementation (20 g/day for six days) as compared to control did not improve hand function (50). Finally, administration of vitamin D analog (4 *µ*g/day) increased lower limb bone mineral density (BMD) at six months to 24 months as compared with placebo administration (both groups received calcium (1.3 g/day) and vitamin D (800 IU/day; 20 *µ*g/day)), accompanied with urinary N-telopeptide, a marker of bone resorption, reduction in the treatment group and no change in markers of bone formation (51).

### Functional Independence and Neurological Recovery

We identified five RCTs classified as Level 1 & 2 studies exploring the effectiveness of dietary interventions/dietary supplements on motor/sensory function, nerve velocity, and functioning score (Supplemental table 3). Two studies focused on individuals with recent injuries, and three included individuals with chronic SCI.

Sixty-four individuals with acute injury were randomized to receive intramuscular injection of 0.5 mg/kg progesterone twice daily and 5µg/kg oral vitamin D twice daily for up to 5 days or placebo (in addition to methylprednisolone on admission according to standard protocol). In both groups, motor and sensory function improvement was observed at six months since the initiation of treatment. Those who received progesterone and vitamin D had significantly higher sensory and motor American Spinal Injury Association Impairment Scale (AIS) score after six months of therapy. In the progesterone and vitamin D group, the AIS motor and sensory scores were significantly higher in those receiving the therapy within the first four hours after injury compared to those receiving it after four hours (52). A small safety and feasibility pilot study (n=7) assessed the effects of a ketogenic diet (i.e., 70–80% total energy as fat) and standard diet on the neurological recovery of recently injured individuals. Individual upper extremity motor scores increased over intervention period in the ketogenic diet group, whereas no change was observed in the standard diet group. No significant changes in sensory outcomes were observed (48). An RCT exploring the effectiveness of intermittent fasting on motor and sensory function did not show significant improvements comparing pre- vs. post-intervention periods (43). Other RCT explored the effectiveness of 435 mg of docosahexaenoic acid and 65 mg of eicosapentaenoic acid capsules daily on Functional Independence Measure and Functional Assessment Measure (FIM+FAM) scale in individuals with chronic SCI. After 14 months of intervention, no significant changes in those scores were observed with omega-3 fatty acids intervention (53). Similarly, a 12-week anti-inflammatory diet program, despite a significant reduction in inflammation in the treatment group, was not associated with changes in peripheral motor nerve conduction (54).

### Urinary system

We identified 7 interventional studies (6 RCTs and one pre-post study) reporting on the effectiveness of cranberry-derived supplements and probiotics on urinary tract infection (UTI)-related outcomes. Six were classified as Level 1 & 2 and one as Level 4 study (Supplemental Table 3).

Based on the findings of the Spinal-injured neuropathic bladder antisepsis (SINBA) trial, which is currently the largest in the field (n=305), individuals with neurogenic bladder and stable bladder management receiving 1600 mg cranberry supplement for six months did not have significantly longer UTI-free period as compared to placebo (55). Similarly, intervention with concentrated cranberry extract (2g/day capsule) in a population of catheter-free community-dwelling individuals with pre-existing bacteriuria did not reduce the bacteriuria (≥ 104 colonies /mL urine), pyuria (≥10 urinary leukocyte/*µ*L urine), and did not prevent symptomatic UTI over 6 months (56). A parallel RCT among veterans with neurogenic lower urinary tract dysfunction and positive urine bacterial colonization (≥50 K CFU/ml) compared the cranberry extract rich in proanthocyanidins (PACs, 36mg/capsule) and placebo intervention for two weeks. There was no reduction of bacteriuria and pyuria with concentrated PACs. This study was terminated prematurely due to difficulties with recruitment and funding and was underpowered to study the primary outcome (57). Two cross-over trials showed conflicting findings. Linsenmeyer et al. (58) reported no difference in bacterial or leukocyte counts with 1'400mg/day cranberry supplement in a 4-week study. On the contrary, in a study among veterans that lasted six months, Hess et al. did not find differences in incidence of bacteriuria between cranberry and placebo groups, yet, fewer UTI during the cranberry period were identified. In addition, they reported that one's glomerular filtration rate (GFR) might influence the effectiveness of cranberry supplements via interplay between bacterial adherence inhibition and high GFR (> 75 ml min−1), which work together to eliminate pathogens (59).

We identified a single RCT comparing the effectiveness of probiotic therapy in preventing UTI among SCI individuals with stable neurogenic bladder management. Authors concluded that none of the applied probiotics RC14-GR1 (Lactobacillus reuteri RC-14+Lactobacillus GR-1) or LGG-BB12 (Lactobacillus rhamnosus GG+Bifidobacterium BB-12) did not influence the risk of UTI. Nevertheless, they speculate that using RC14-GR1 alone may be beneficial (the Kaplan–Meier survival curves appeared to show longer UTI-free survival for RC14-GR1 compared to other groups) (60). The authors, however, acknowledge that they failed to recruit targeted sample size, and thus, the results should be interpreted with caution. In another publication from the same trial, they reported that probiotics were ineffective in clearing multi-resistant organisms, yet, they speculate that RC14-GR1 is effective at preventing new colonization with multi-resistant gram-negative organisms as compared to placebo group (61).

### Gastrointestinal System

We identified two Level 2 trials exploring the benefits of dietary intervention on gastrointestinal health, Supplemental table 3. An RCT explored the effectiveness of a low-carbohydrate, high-protein diet (LC/HP diet) on gut microbiome composition among individuals with glucose intolerance. After eight weeks, significant changes in alpha- and beta-diversity were observed in LC/HP diet group, whereas no significant differences were observed among participants in the control group which followed the usual diet. Besides, the changes in the following taxa, including increased Bacteroides thetaiotaomicron, Coprococcus, Fusicatenibacter, Tannerellaceae, and decreased Tyzzerella, Phascolarctobacterium, Romboutsia, Clostridium sensu stricto 1, Hungatella, Ruminococcus gauvreauii, family XI, and Bacillales were observed among participants in the diet group. No significant changes were observed in the control group (62). Another trial explored the role of probiotics containing 6.5 × 109 live Lactobacilluscasei Shirota in preventing development of antibiotic-associated diarrhea (AAD), defined as more than two liquid stools per day for more than three days. They reported reduced incidence of AAD in hospitalized SCI patients in the intervention (17.1%) as compared to the control group (54.9%) (63).

### Cognition, Mood and Well-being

We identified one Level 2 RCT reporting on the effectiveness of anti-inflammatory diet on cognition depression scores and a single Level 4 study reporting on the effectiveness of diet and exercise intervention on general well-being (64), Supplemental table 3. Allison et al., tested the hypothesis that an anti-inflammatory diet to reduce inflammation would lead to corresponding changes in neuroactive compounds and improvements in mood and depressive symptoms. Symptoms of depression were assessed using the Center for Epidemiological Studies Depression Scale (CES-D). The CES-D scores decreased in the treatment group from both baseline to 1 month, as well as from baseline to 3 months, while a decrease was observed in control group from baseline to 1 month and no significant change from baseline to 3 months was reported. In addition, those with CES-D scores indicating depression (>16) had concentrations of IL-1β, which were 73 % higher compared to those with lower (<16) CES-D scores. 65 Authors reported no changes verbal learning and memory indices within the observation period (in separate publication based on the same RCT) (41). The authors further explored diet adherence a year post-intrevention. Simillary to trends in other populations, they showed a significant reduction in anti-inflammatory diet compliance (92.6% at 12 months versus 43.0% at the end of the trial). Further, improvement in mood and some components of neuropathic pain observed at the end of the initial intervention were lost at follow-up (12 months after cessation of anti-inflammatory diet intervention), Supplemental table 3 (66).

### Habitual Diet and Health Following Spinal Cord Trauma

Among 11 studies (all being classified as Level 3&4), 9 focused on cardiometabolic risk factors, one on neuropathic pain and one was a post-intervention follow-up study exploring the adherence to anti-inflammatory diet a year post-intervention (Table 2).

A prospective cohort study from the Nethlernads explored the association between lifestyle factors (based on questionnaire on self-care) and CVD risk factors within five years after discharge from inpatient SCI rehabilitation. The question related to physical fitness maintenance was associated with all lipid profiles, while self-care questions regarding low-fat diet were not associated with lipid profiles nor BMI (67). The total healthy eating index (HEI-2015), which is a composite score reflecting the intake of 13 dietary components, was moderately associated with better fasting glucose concentrations in a cross-sectional study (for every 10-point increase of the HEI-2015 a 3.3-mg/dL decrease in fasting glucose was observed) (68). A prospective cohort study including more than 2,000 individuals with SCI studied the association between six behavioral domains (prescription medication usage, alcohol use, smoking, two nutrition factors, and fitness) and all cause and cause-specific mortality (69). Higher smoking and score of prescription medication, and lower fitness levels were associated with higher hazard ratio of dying due to any cause. The nutrition (eating healthy and unhealthy) and alcohol factors were not statistically significant predictors of mortality. Eating mostly unhealthy eating practices nutrition factor was inversely associated with deaths due to heart and blood vessel diseases (HR = 0.94; 95% CI [0.89–0.99]) (69). In a case-control study, comparing dietary habits among age, sex, and race-matched SCI and able-bodied individuals (ABI), the SCI group consumed less fruit, dairy, and whole grains and more meat (vegetable intake was similar). No significant associations were observed between food groups and CVD risk factors in any of the two comparison groups. When looking at whether the association between food groups and CVD risk factors was different between SCI and ABI, a significant interaction term (group status x food group) was observed for whole-grain intake and glucose, hs-CRP and systolic and diastolic blood pressure and between fruit and vegetable intake and insulin levels (70). Dietary fat and carbohydrate intake were negatively associated with cardiometabolic risk factors (71, 72, 73). Studies focusing on micronutrient intake explored the role of amino acids and vitamin D intake on cardiometabolic risk factors. Some amino acids had positive some had negative associations with cardiometabolic risk factors (72), while, individuals who reported higher vitamin D intake (an average intake of 5.33 ± 4.14 mcg) may have better lipid and glucose profile as compared to individuals with lower vitamin D dietary intake (0.74 ± 0.24 mcg) (74). In cross-sectional study among Iranian men with one increase in the dietary inflammatory index (DII) was significantly associated with higher scores of depressive symptoms and higher odds of having at least mild depressive symptoms; while no significant association was found between the DII and anxiety and stress (75).

## Discussion

**Figure 5:**
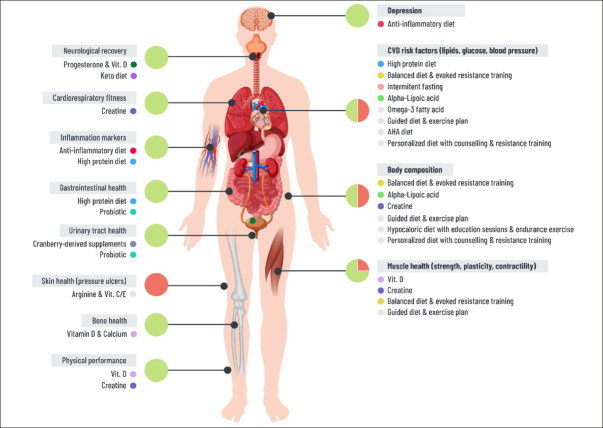
Graphical summary of potentially beneficial dietary interventions

This systematic review has mapped the most promising dietary strategies linked with favourable changes in health and well-being in SCI population (graphical summary provided in Figure 5). In brief, based on Level 1 and 2 studies: (i) high protein diet, intermittent fasting, balanced diet in combination with physical conditioning and electrical stimulation and alpha-lipoic acid improved cardiometabolic risk factors; (ii) creatine and vitamin D supplements improved musculoskeletal health, cardiorespiratory fitness, and physical performance; (iii) cranberry-derived supplements and probiotics improved some aspects of gastrointestinal and urinary systems, and (iv) limited evidence supported benefits of progesterone and vitamin D, and keto diet intervention in improving aspects of neurological recovery. Herewith we discuss the most critical findings, emphasize literature gaps, and provide directions for future research.

This figure depicts only dietary interventions which were shown to be beneficial. The overview of interventions which were not beneficial is provided in Supplemental table 2 & 3. Coloured circles indicate Level 1 and Level 2 studies. Grey colored circles indicate Level 3 and Level 4 studies. Large Green/Red circles depict the ratio between Level 1/ 2 (green) vs Level 3/ 4 studies (red color).

Most studies identified through our systematic review linked dietary interventions with changes in cardiometabolic risk profile. First, a high-protein diet improved glucose homeostasis parameters, body morphology, and inflammation markers (40) and caused beneficial changes in microbiome diversity (taxa involved in fibre metabolism were increased, while those linked to CVD and metabolic disorders were reduced) (62). Dietary carnitine and choline ingestion (originating from animal protein sources) can lead to significant elevations in trimethylamine N-oxide (TMAO), which has been shown to have several adverse effects on host metabolism, particularly those that affect cardiovascular health (76). In contrast, anaerobic fermentation of undigested nutrients (resistant starch, dietary fibre) produces short-chain fatty acids (SCFAs), which have been linked to decreased CVD risk (77). These trials did not provide measurements of biologically active molecules generated by the gut microbiota. Future studies are to replicate their findings and explore changes in metabolic markers following a protein-rich diet, which is of particular interest for individuals with impaired glucose tolerance. Second, a small RCT indicated more advantageous changes in cardiometabolic risk profile among those who followed a balanced diet and were involved in evoked resistance training with neuromuscular electrical stimulation than those who followed a nutrition plan only (42). Considering that lifestyle monotherapies such as physical activity or dietary interventions solely are likely insufficient to modify cardiometabolic risk factors in persons with SCI (e.g., those with injury level above T6) (78, 79), future trials should compare the effectiveness of dietary interventions alone and in combination with physical activity regimes with and without functional electrical stimulation using a proper control group (preferably placebo). Third, in the study by Allison et al. (41), anti-inflammatory diet in addition to supplements with high antioxidant potential (e.g., vitamins and omega-3 fatty acids) improved inflammatory status. The dietary regime used in the trial was similar to the Mediterranean diet (e.g., higher consumption of fruits and vegetables, olive oil, fish, whole grains, and tree nuts and reduction of red/processed meats and refined sugars); however, the addition of micronutrients and vitamins with high antioxidant potential make the interpretation of findings challenging. In particular, the change in vitamin A, carotenoids, omega-3, and zinc over the intervention period was negatively correlated with several pro-inflammatory mediators (41). Future studies are to explore which micronutrients may play the major role in improving inflammatory profile among SCI individuals. Finally, alpha-lipoic acid improved glucose homeostasis, body composition, and blood pressure, which is in line with a recent meta-analysis indicating its modest anti-obesity and anti-diabetic properties in general population (80, 81). No benefits on inflammation status were observed; nevertheless, study participants were otherwise healthy men with SCI which may have affected the null findings.

We identified a few studies reporting on neurological recovery, musculoskeletal health, and physical performance. Among those, limited evidence supported benefits of progesterone and vitamin D therapy on neurological recovery. While other trials using vitamin D in SCI individuals yield contradicting results, a study supplementing vitamin D analog (1-alpha-hydroxyvitamin D(2) [1-alpha D(2)]) improved lower limb BMD in chronic SCI (51), whereas another trial providing n-3 polyunsaturated fatty acids with calcium and vitamin D did result in any significant improvements in pro-inflammatory cytokines in individuals with SCI and osteoporosis (BMD was not assessed in this study) (47).

The role of vitamin D supplements in bone health has been questioned over the past few years. Recent Mendelian randomization studies showed that genetically determined vitamin D was not causally associated with bone mineral density in the general population; however, these studies are based on general population, and vitamin D may still improve bone health in high-risk populations (82, 83). Thus, further RCTs are warranted to investigate the role of vitamin D supplementation in both subacute (within rehabilitation treatment modalities) and chronic injury phases (among those with osteoporosis or vitamin D deficiency). In addition, creatine supplementation improved exercise capacity in persons with cervical injuries, and future studies are to explore the role of combined creatine and physical exercise intervention on functioning outcomes among those with high injury levels.

Further, the evidence on the prophylactic role of cranberry-derived supplements on UTI risk remains inconclusive. Contradicting findings may be driven by differences in the quality of supplements (e.g., only a single trial used standardized PACs rich product, whereas others did not provide PAC content), duration of the intervention (2 weeks to 6 months), underlying comorbidities (stable management vs. individuals with bacteriuria) and bladder management (indwelling, intermittent, or reflex voiding with or without external/condom catheter). Some authors advise a crossover study design to overcome high heterogeneity in the underlying population; nonetheless, the major challenges remain in choosing the optimal washout period duration and seasonality of UTI occurrence. We advise a parallel study design focusing on high-risk population (those with recurrent UTI) and considering intermediate outcomes such as plasma or urinary markers of inflammation and changes in urinary microbiome rather than focusing on incident UTI. Moreover, as acknowledged in recent SCI-specific guidelines, factors such as obesity, smoking, excessive caffeine, alcohol or water consumption, may worsen the symptoms of overactive bladder, and future trials should provide a detailed assessment of relevant lifestyle factors which may influence the effectiveness of cranberry supplements (84). Finally, the use of probiotics, although promising, has rarely been studied in SCI population, and its benefits beyond the effect on gut microbiome merit additional research (e.g., metabolic or inflammation markers).

### Strengths and Weaknesses

This is the most comprehensive overview of the current body of evidence on the role of habitual diet and dietary interventions in prevention of SHC among SCI population. A highly sensitive search strategy was used and reference lists of included articles were hand searched to identify as many relevant studies as possible and reduce the risk of publication bias. Mapping most promising dietary interventions was based on the strength of the study design and methodological quality assessment, whereas the evidence of poorer methodological quality was provided only for transparency.

Yet, our review has some weaknesses due to methodological limitations of underlying evidence, which has to be considered and taken into context when interpreting our results. First, the major methodological issues in clinical trials referred either to lack of randomization, small sample size (no power calculation), or absence of study personnel blinding. Among 32 clinical trials, nine were pilot studies with the primary objective defined as safety and feasibility of dietary interventions. Pilot studies are usually underpowered to achieve statistical significance at the commonly used 5% level. They should focus on descriptive statistics and estimation, using confidence intervals rather than formal hypothesis testing (85). Despite this, many trials still quote and interpret P-values, and their results should be interpreted cautiously. Furthermore, we could not pool the data together through meta-analysis since the intervention duration, type of dietary intervention/supplement, and control groups varied considerably across trials. Yet, to depict the magnitude of effect across individual trials we provided the forest plots depicting the mean changes between intervention and control group (Figure 3) and before vs. after intervention (Figure 4 a & b). Further, due to significant clinical heterogeneity, it was difficult to determine whether any personal or injury characteristics had an impact on the effectiveness of dietary interventions. Among the eleven observational studies, two were longitudinal, while six reported only cross-sectional associations between diet and health outcomes. Besides, being at risk of reverse causality bias, these studies provided mostly mean values (SDs) of cardiometabolic biomarkers among comparison groups without providing adjustments for potential confounders. Therefore, all observational studies were evaluated as Level 3 or Level 4 studies indicating low certainty of evidence.

Second, women were underrepresented across the studies due to lower rate of females affect by injury and purposive exclusion for women in research (86). Sex (and gender) may influence both the exposure (habitual diet and underlying lifestyle patterns) and outcomes (cardiometabolic diseases and UTI risk, among others); thus, our findings may be mainly generalized to men with SCI, and future studies are to explore the modifying role of sex in the effectiveness of potentially beneficial dietary approaches as mapped within this review. Further, the mean age across intervention studies varied between 23 years to 57 years, while injury duration ranged from only a few days to on average 24 years (Figure 2). A complex interplay between age at injury, injury duration and age-related physiological changes, body composition shifts, and health considerations should be considered in future research and when designing dietary interventions for older SCI individuals. Third, the majority of studies (84.4%) focused on those with chronic injury (≥12 months months) and recruited otherwise healthy SCI individuals, which may minimize the magnitude of change among blood biomarkers and may have influenced the null findings observed for some outcomes (e.g., body composition or blood lipids). Fourth, only a single trial studied diet adherence a year post-intervention and reported a significant reduction in anti-inflammatory diet compliance at follow-up (66). In addition, the quality of observational studies exploring the role of habitual diet was limited (only Level 3 and 4 studies were identified), which further limits us in understanding the role of long-term adherence to healthy diet patterns. Adherence to healthy diet and healthy lifestyle is a challenge, and future quantitative and qualitative, observational and intervention studies, should identify the most important facilitators and barriers specific to SCI population to enhance healthy lifestyle adherence as a key modifiable risk factor for developing SHC in this vulnerable population. Finally, based on evidence originating from Level 3 and 4 studies omega-3 fatty acids supplements and AHA diet were linked with improvement in cardiometabolic risk factors. However, due to methodological limitations of clinical trials, these findings should be replicated within a well-designed clinical trial with sufficiently long follow-up.

## Conclusions

This review focuses on the role of dietary interventions and habitual diet in health and disease among SCI population. The evidence of moderate quality supports the beneficial role of dietary interventions alone (i.e., alpha-lipoic acid supplementation, creatine and vitamin D supplementation, high protein diet, anti-inflammatory diet) and personalized diet in combination with physical exercise and neuromuscular electrostimulation on improving cardiometabolic and musculoskeletal health and physical performance, post-injury. Currently, the evidence regarding the benefits of cranberry-derived dietary supplements in UTI prevention and the benefits of probiotic interventions in improving bowel function remains scarce. It is critical to emphasize that our findings were based on a limited number of RCTs. Within this systematic review, we have provided an overview of the most promising dietary interventions in preventing major SHC post-injury. Further research is warranted to develop timely and effective preventive strategies targeting major SCH in aging SCI (e.g., cardiometabolic disease, UTI, osteoporosis). We hope that this systematic review helps guiding future endeavours examining the efficacy of nutrition and exercise in improving the health status and overall well-being of individuals with SCI.

## Electronic Supplementary Material

Supplementary material, approximately 327 KB.

*Author Contributions:* MG, CP, IEH and SR obtained the funds and initiated the study. MG supervised the study. BM created the search strategy. SS, MG, LGK, MB, CD, EV, MB, AS, ND, JS, SR, CP, IEH participated in the literature search, data collection and interpretation. SS wrote the first draft of the manuscript. All authors were involved in revising the draft manuscript and approving the final content.

*Funding:* This work is supported by the Swiss Paraplegic Foundation, Nottwil, Switzerland (Project ID: Foko_2021_01). Open Access funding provided by University of Bern.

*Data Availability Statement:* The datasets generated during the current review are available in the online supplement.
