# Supplementary material for: Mapping of Dietary Interventions Beneficial in the Prevention of Secondary Health Conditions in Spinal Cord Injured Population: A Systematic Review
Source: J Nutr Health Aging. 2024 Jan 4;27(7):524–41. doi: 10.1007/s12603-023-1937-6 (PMC12929980; doi:10.1007/s12603-023-1937-6)
Supplement: Supplementary file 1 — Supplementary material, approximately 327 KB. [file mmc1.docx]

**ONLINE SUPPLEMENT**

**Mapping of dietary interventions beneficial in the prevention of secondary health conditions in spinal cord injured population: a systematic review**

Stevan Stojic^1^, Inge Eriks-Hoogland^1,2^, Magda Gamba^3,4^, Ezra Valido^1,2^, Beatrice Minder^5^, Angéline Chatelan^6^, Leonidas G. Karagounis^3,7^, Monica Ballesteros^3^, Catalina Díaz^3^, Mirjam Brach^1^, Jivko Stoyanov^1^, Nicola Diviani^1,2^, Sara Rubinelli^1,2^_,_ Claudio Perret^1,2^¥, Marija Glisic^1,3^¥

**¥ denotes equal contribution**

^1^ Swiss Paraplegic Research, Nottwil, Switzerland

^2^Faculty of Health Sciences and Medicine, University of Lucerne, Lucerne, Switzerland

^3^Institute of Social and Preventive Medicine, University of Bern, Switzerland

^4^Graduate School for Health Sciences, University of Bern, Bern, Switzerland

^5^Public Health and Primary Care Library, University Library of Bern, University of Bern, Bern, Switzerland

^6^Department of Nutrition and Dietetics, Geneva School of Health Sciences, HES-SO University of Applied Sciences and Arts Western Switzerland, Geneva, Switzerland

^7^Mary MacKillop Institute for Health Research (MMIHR), Australian Catholic University (ACU), Melbourne, Australia

Contents

[Supplemental Table 1. Eligible study designs and criteria for classifying the level of evidence for individual studies. 3](#_Toc136524502)

[Supplemental table 2. Summary of findings from interventional studies on cardiometabolic risk factors, cardiorespiratory fitness, musculoskeletal health, and physical performance 4](#_Toc136524503)

[Supplemental table 3. Summary of findings from interventional studies on other health outcomes 7](#_Toc136524504)

[Supplemental Table 4. Quality Assessment of Controlled Intervention Studies 8](#_Toc136524505)

[Supplemental Table 5. Quality Assessment Tool for observational studies 11](#_Toc136524506)

| Supplemental Table 1. Eligible study designs and criteria for classifying the level of evidence for individual studies. | | | |
| --- | --- | --- | --- |
| **Level of evidence** | **Study design** | **Study quality score based on NIH** | **Description study design** |
| **Level 1 study** | Randomized controlled trial | High methodological quality | Using within-subjects comparison with randomized conditions or cross-over designs |
| **Level 2 study** | Randomized controlled trial | Moderate methodological quality | Using within-subjects comparison with randomized conditions or cross-over designs |
|  | Non-randomized controlled trial | High methodological quality | Comparing intervention vs. control groups (not randomly allocated) |
|  | Prospective cohort study | High methodological quality | Longitudinally comparing at least two similar groups (one exposed one unexposed) |
|  | Case-control study | High methodological quality | Using a case-control study design to compare two types of diet/dietary patterns |
| **Level 3 study** | Randomized controlled trial | Low methodological quality | Using within-subjects comparison with randomized conditions or cross-over designs |
|  | Non-randomized controlled trial | Moderate methodological quality | Comparing intervention vs. control groups (not randomly allocated) |
|  | Prospective cohort study | Moderate methodological quality | Longitudinally comparing at least two similar groups (one exposed one unexposed) |
|  | Retrospective cohort study | High or moderate methodological quality | Retrospectively comparing an exposed/interventional group to a historical control group |
|  | Case-control study | Moderate methodological quality | Using a case-control study design to compare two types of diet/dietary patterns |
|  | Pre-post study | High methodological quality | Using a baseline measure, intervention and a post-test in a single group |
| **Level 4 study** | Non-randomized controlled trial | Low methodological quality | Comparing intervention vs. control groups (not randomly allocated) |
|  | Prospective cohort study | Low methodological quality | Longitudinally comparing at least two similar groups (one exposed one unexposed) |
|  | Retrospective cohort study | Low methodological quality | Retrospectively comparing an exposed/interventional group to a historical control group |
|  | Case-control study | Low methodological quality | Using a case-control study design to compare two types of diet/dietary patterns |
|  | Pre-post study | Low or moderate methodological quality | Using a baseline measure, intervention and a post-test in a single group |
|  | Cross-sectional study | Regardless of the methodological quality | Comparing two groups exposed and unexposed to specific diet/micro- or macronutrient |

# Supplemental table 2. Summary of findings from interventional studies on cardiometabolic risk factors, cardiorespiratory fitness, musculoskeletal health, and physical performance

| **Outcome** | **No. of studies** | **Pre- to post-intervention effect within intervention group** | | **Pre- to post-intervention effect between intervention and control group** | | **Level of evidence** |
| --- | --- | --- | --- | --- | --- | --- |
|  |  | **Beneficial** | **Zero** | **Beneficial** | **Zero** |  |
| **Cardiometabolic risk factors** | **16** |  |  |  |  |  |
| Glucose homeostasis | 9 | High protein diet**^1^;** Ketogenic diet**^2^;** Balanced diet and  Resistance  Training with neuromuscular electrical stimulation; Balanced diet**^3^**; Individualized exercise and nutrition plans**^4^;** alpha-lipoic acid**^5^;** Intermittent fasting**^6^** | Guided diet & exercise plan**^7^;** Personalized diet with counselling &  Resistance  Training**^8^;** n-3 fatty acids**^9^** | Balanced diet and  Resistance  Training with neuromuscular electrical stimulation**^3^;** alpha-lipoic acid**^5^** |  | Level 1**^5^**  Level 2**^1-3 6^**  Level 4**^4 7-9^** |
| Blood lipids | 8 | Hypocaloric diet with Education sessions & endurance exercise; Balanced diet and  Resistance  Training with neuromuscular electrical stimulation**^3^;**AHA diet^10^ | High protein diet**^1^;** Ketogenic diet**^2^;** Nutrition education & Personalized diet **^11^;** Balanced diet**^3^**; Guided diet & exercise plan**^7^;** n-3 fatty acids**^9^;** Individualized exercise and nutrition plans**^4^** | Balanced diet and  Resistance  Training with neuromuscular electrical stimulation**^3^** | Nutrition education & Personalized diet **^11^** | Level 2**^1-3 11^**  Level 4^4 7 9 10^ |
| Blood pressure | 5 | Guided diet & exercise plan**^7^;** alpha-lipoic acid**^5^;** n-3 fatty acids**^12^** | Hypocaloric diet with Education sessions & endurance exercise**^13^;** Nutrition education & Personalized diet **^11^** | alpha-lipoic acid**^5^** |  | Level 4**^7 12 13^**  Level 2**^11^**  Level 1**^5^** |
| Anthropometrics | 6 | Hypocaloric diet with Education sessions & endurance exercise**^13^;**Personalized diet with counselling &  Resistance  Training**^8^;** Individualized exercise and nutrition plans**^4^'** alpha-lipoic acid**^5^;** Intermittent fasting**^6^** | Balanced diet and  Resistance  Training with neuromuscular electrical stimulation; **^3^** Balanced diet**^3^;** Nutritional counselling alone**^8^** | Personalized diet with counselling &  Resistance  Training**^8^'** alpha-lipoic acid**^5^** | Nutrition education & Personalized diet **^11^;** Balanced diet and  Resistance  Training with neuromuscular electrical stimulation**^3^** | Level 4**^8 13^**  Level 2**^3 6 11^**  Level 1**^5^** |
| Body composition | 5 | High protein diet**^1^;** Balanced diet and  Resistance  Training with neuromuscular electrical stimulation**^3^;** Personalized diet with counselling &  Resistance  Training**^8^;**Creatine **^14^** | Hypocaloric diet with Education sessions & endurance exercise**^13^;** Balanced diet; Nutritional counselling alone**^8^** | Personalized diet with counselling &  Resistance  Training**^8^;** Creatine **^14^** | Balanced diet and  Resistance  Training with neuromuscular electrical stimulation**^3^** | Level 2**^1 3 14^**  Level 4**^8 13^** |
| Inflammation markers | 3 | High protein diet**^1^;** anti-inflammatory diet**^15^** | MorDHA capsules**^16^** | anti-inflammatory diet**^15^** |  | Level 2**^1 15^**  Level 1**^16^** |
| **Cardiorespiratory fitness** | 2 |  |  |  |  |  |
| Peak power output | 1 | Creatine^17^ |  | Creatine^17^ |  | Level 2^17^ |
| Peak VO2 (oxygen uptake) | 2 |  | Creatine^17^  Diet & Excersise^7^ | Creatine^17^ |  | Level 2^17^  Level 4^7^ |
| **Muscle Health** | 4 |  |  |  |  |  |
| Handgrip strength | 3 | Guided diet & exercise plan^7^ | Vit D^18^ | Creatine^14^ | Vit D^14^ | Level4^7 18^  Level 2^14^ |
| Maximal voluntary wrist extensor isometric contraction strength and endurance | 1 |  |  |  | Creatine^19^ | Level 2^19^ |
| Arm muscle area | 1 | Vit D^14^; Creatine^14^ |  | Creatine^14^ | Vit D^14^ | Level 2^14^ |
| Skeletal  muscle CSA | 1 | Balanced diet & RT **^3^** | Diet**^3^** | Balanced diet & RT **^3^** | Diet **^3^** | Level 2**^3^** |
| **Physical performance** | 3 |  |  |  |  |  |
| 20-m sprint | 2 |  | Vit D^18^ |  | Vit D^14^; creatine^14^ | Level 4^18^  Level 2^14^ |
| Manual wheelchair slalom test | 1 |  | Vit D^14^; creatine^14^ |  | Vit D^14^; creatine^14^ | Level 2^14^ |
| Seated  medicine ball throw | 1 | Vit D^14^ ;Creatine^14^ |  |  | Vit D^14^; creatine^14^ | Level 2^14^ |
| Chest press | 1 | Vit D^14^; Creatine^14^ |  |  | Vit D^14^; creatine^14^ | Level 2^14^ |
| Triceps | 1 | Creatine^14^ | Vit D^14^; |  | Vit D^14^; creatine^14^ | Level 2^14^ |
| Pec deck | 1 | Creatine^14^ | Vit D^14^; |  | Vit D^14^; creatine^14^ | Level 2^14^ |
| Lat pulldown | 1 | Creatine^14^ | Vit D^14^; |  | Vit D^14^; creatine^14^ | Level 2^14^ |
| Grasp and Release Test | 1 |  |  |  | Creatine^19^ | Level 2^19^ |
| **Bone health** | 2 |  |  |  |  |  |
| Bone mineral density | 2 | Vit D &Calcium ^20^ | Diet, education & exercise**^13^** | ---- | ---- | Level 2 ^20^  Level 4**^13^** |
| Urine NTx | 1 | Vit D &Calcium ^20^ | ---- | ---- | ---- | Level 2 ^20^ |

# Supplemental table 3. Summary of findings from interventional studies on other health outcomes

| **Outcome** | **No. of studies** | **Pre- to post-intervention effect within intervention group** | | **Pre- to post-intervention effect between intervention and control group** | | **Level of evidence** |
| --- | --- | --- | --- | --- | --- | --- |
|  |  | **Beneficial** | **Zero** | **Beneficial** | **Zero** |  |
| **Functional independence/neurological recovery** | 4 |  |  |  |  |  |
| Motor and sensory function | 3 | Keto diet**^2^** | Intermittent fasting^6^; | Progesterone + Vit D ^21^ | ---- | Level 1^21^  Level 2^2 6^ |
| FIM &FAM score | 1 |  | MorDHA^22^ | --- | ---- | Level 1^22^ |
| Motor NCV (nerve conduction velocity) | 1 |  | Anti-inflammatory diet **^23^** |  |  | Level 2**^23^** |
| M-wave amplitude | 1 |  | Anti-inflammatory diet**^23^** |  |  | Level 2**^23^** |
| **Skin health** | 1 |  |  |  |  |  |
| Pressure ulcers healing | 1 | ---- | ---- | Arginine & Vit C/E^24^ | ---- | Level 3^24^ |
| **Urinary tract** | 6 |  |  |  |  |  |
| Urine biofilm load /bacterial count | 4 | Cranberry juice^25^; | Concentrated proanthocyanidins in the cranberry supplement^26^---- |  | 400-mg cranberry tablet ^27^; 2g-cranbery capsule ^28^; Concentrated proanthocyanidins in the cranberry supplement^26^ | Level 2^27^, ^28^ ^26^  Level 4 ^25^ |
| Urine pH | 2 | ---- | ---- | ---- | 400-mg cranberry tablet ^27^; 2g-cranbery capsule ^28^ | Level 2^27^, ^28^ |
| Urinary WBC count | 1 | ---- | ---- | ---- | 400-mg cranberry tablet ^27^ | Level 2^27^ |
| Symptomatic UTI episodes | 3 | Cranberry tablet^29^ |  | 2g cranberry capsule^28^ Cranberry tablet^29^ | Probiotic^30^; 1600 mg cranberry tablet^31^ | Level 1^30^ ^31^  Level 2^28^; ^29^ |
| **Gastrointestinal health** | 2 |  |  |  |  |  |
| Gut microbiome diversity | 1 | Low-carbohydrate, high-protein^32^ | Usual diet^32^ | ----- | ----- | Level 2^32^ |
| Antibiotic-induced diarrhoea | 1 | Probiotic ^33^ | ----- | ----- | ----- | Level 2^33^ |
| **Other** |  |  |  |  |  |  |
| **General well-being and readiness to change** | 1 | ----- | Diet & Excersise^7^ | ------ | ----- | Level 4^7^ |
| **Depression** | 2 |  |  |  |  |  |
| CES-D score | 1 | Anti-inflammatory diet **^34^** | ----- | Anti-inflammatory diet **^34^** | ---- | Level 2**^34^** |
| **Cognitive function** |  |  |  |  |  |  |
| California Verbal Learning Test (CVLT scores) | 1 |  | Anti-inflammatory diet Allison 2017 **^15^** |  |  | Level 2**^15^** |
|  |  |  |  |  |  |  |

# Supplemental Table 4. Quality Assessment of Controlled Intervention Studies

| **Lead Author, Publication Year** | **Study Design** | **1** | **2** | **3** | **4** | **5** | **6** | **7** | **8** | **9** | **10** | **11** | **12** | **13** | **14** | **Methodological quality ^1^** | **Study Level** |
| --- | --- | --- | --- | --- | --- | --- | --- | --- | --- | --- | --- | --- | --- | --- | --- | --- | --- |
| Dolbow et al, 2021 ^8^ | NRCT | N | N | N | N | N | Y | N | NR | Y | Y | Y | NR | Y | N | Moderate (5/14, 36%) | 3 |
| Szlachcic et al., 2001^10^ | NRCT | N | N | N | N | N | NR | Y | NR | Y | Y | Y | N | Y | NA | Low (5/14, 35.7%) | 4 |
| Brewer et al, 2010 ^24^ | NRCT | N | N | N | N | N | Y | Y | Y | Y | Y | Y | NR | Y | N | Moderate (7/14, 50%) | 3 |
| Radomski et al., 2011^7^ | Pre-post | Y | Y | Y | N | NR | Y | Y | NR | Y | Y | N | NA | ---- | ---- | Moderate (7/12, 58.3%) | 4 |
| Chen et al., 2006^13^ | Pre-post | Y | Y | N | NR | NR | Y | Y | T | Y | Y | N | NA | ---- | ---- | Moderate (7/12, 58.3%) | 4 |
| Javierre et al., 2005^9^ | Pre-post | Y | Y | Y | N | NR | Y | Y | NR | Y | Y | N | NA | ---- | ---- | Moderate (7/12, 58.3%) | 4 |
| Myers, et al., 2012^4^ | Pre-post | Y | Y | Y | Y | NR | Y | Y | NR | Y | Y | Y | Y | N | N | Moderate (10/14, 71.4%) | 4 |
| Reid et al., 2015^25^ | Pre-post | Y | N | Y | Y | N | Y | Y | NR | N | Y | Y | NR | ---- | ---- | Moderate (7/12, 58.3%) | 4 |
| Pritchett et al., 2015^18^ | Pre-post | Y | N | Y | Y | N | Y | Y | NR | N | Y | Y | NR | ---- | ---- | Moderate (7/12, 58.3%) | 4 |
| Javierre et al., 2006^12^ | Pre-post | Y | Y | Y | Y | N | Y | Y | NR | Y | Y | Y | NR | --- | --- | Moderate  (9/12, 75%) | 4 |
| Allison et al. 2015 ^34^ | RCT | Y | Y | NR | N | NR | Y | Y | Y | Y | Y | Y | N | Y | NR | Moderate (9/14, 64%) | 2 |
| Gorgey et al., 2012^3^ | RCT | Y | Y | NR | NR | NR | Y | Y | Y | Y | Y | Y | N | Y | NR | Moderate (9/14, 64%) | 2 |
| Li et al., 2018^1^ | RCT | Y | NR | NR | NR | NR | N | N | N | Y | Y | Y | NR | Y | N | Low (5/14, 35.7%) | 3 |
| Mohammadi et al., 2015^5^ | RCT | Y | Y | NR | Y | Y | Y | Y | Y | Y | Y | Y | N | Y | N | High (11/14, 78.6%) | 1 |
| Sabour et al, 2012^16^ | RCT | Y | Y | NR | Y | Y | Y | N | Y | Y | Y | Y | N | Y | Y | High (11/14, 78.6%) | 1 |
| Sabour et al., 2018^11^ | RCT | Y | Y | NR | NR | NR | Y | Y | Y | Y | Y | Y | N | Y | NR | Moderate (9/14, 64%) | 2 |
| Linsenmeyer, et al, 2004^27^ | RCT | Y | NR | NR | Y | Y | NR | N | NR | Y | Y | Y | NR | Y | N | Moderate (7/14, 50%) | 2 |
| Waites et al., 2004^28^ | RCT | Y | NR | NR | Y | Y | N | N | N | Y | Y | Y | NR | Y | N | Moderate (7/14, 50%) | 2 |
| Toh et al., 2019^30^ | RCT | Y | Y | NR | Y | Y | Y | Y | Y | Y | Y | Y | N | Y | N | High (11/14,78.6%) | 1 |
| Bauman et al, 2005 ^20^ | RCT | Y | NR | NR | Y | Y | Y | Y | Y | Y | Y | Y | NR | Y | NR | Moderate (10/14, 71.4%) | 2 |
| Aminmansour et al, 2016 ^21^ | RCT | Y | Y | NR | Y | Y | Y | Y | Y | Y | Y | Y | Y | Y | NR | High (12/14, 85.7%) | 1 |
| Javidan et al., 2014^22^ | RCT | Y | Y | NR | Y | Y | Y | Y | Y | Y | Y | Y | NR | Y | N | High (11/ 14, 78,6%) | 1 |
| Kendall et al., 2005^19^ | RCT | Y | Y | NR | Y | Y | Y | Y | Y | NR | Y | Y | N | Y | N | Moderate (10/14, 71.4%) | 2 |
| Yarar-Fisher et al., 2018^2^ | RCT | Y | NR | NR | N | N | NR | Y | Y | Y | Y | Y | NR | Y | N | Moderate (7/14, 50%) | 2 |
| Amorim et al, 2018 ^14^ | RCT | Y | N | NR | Y | Y | Y | Y | Y | Y | Y | Y | NR | Y | NR | Moderate (10/14, 71.4%) | 2 |
| Li et al., 2022^32^ | RCT | Y | Y | NR | NR | Y | Y | N | Y | Y | Y | Y | NR | Y | NR | Moderate (9/ 14, 64.2%) | 2 |
| Zheng et al., 2021^6^ | RCT | Y | Y | NR | NR | NR | Y | Y | Y | Y | Y | Y | NR | Y | NR | Moderate (9/14, 64.3%) | 2 |
| Jacobs et al., 2002^17^ | RCT | Y | NR | NR | Y | Y | Y | Y | Y | Y | Y | Y | NR | Y | N | Moderate (10/14, 71.4%) | 2 |
| Sappal et al., 2018^26^ | RCT | Y | Y | NR | Y | Y | NR | Y | Y | Y | Y | Y | NR | Y | N | Moderate (10/14, 71.4%) | 2 |
| Hess et al., 2008^29^ | RCT | Y | Y | NR | Y | Y | NR | Y | NR | Y | Y | Y | N | Y | N | Moderate (9/14, 64.3%) | 2 |
| Lee et al., 2007^31^ | RCT | Y | Y | NR | Y | Y | Y | Y | Y | Y | Y | Y | Y | Y | N | High (12/14, 85.7%) | 1 |
| Wong et al., 2013^33^ | RCT | Y | Y | NR | N | N | Y | Y | Y | Y | Y | Y | Y | NA | Y | Moderate (10/14, 71.4%) | 2 |
| Abbreviations: CD, cannot determine; NA, not applicable; NR, not reported  ^1^Methodological quality rating: High ((75-100%), Moderate (50-75%), or Low (<50%))  **Criteria used to asses risk of bias of controlled clinical trials:**  1. Was the study described as randomized, a randomized trial, a randomized clinical trial, or an RCT?  2. Was the method of randomization adequate (i.e., use of randomly generated assignment)?  3. Was the treatment allocation concealed (so that assignments could not be predicted)?  4. Were study participants and providers blinded to treatment group assignment?  5. Were the people assessing the outcomes blinded to the participants' group assignments?  6. Were the groups similar at baseline on important characteristics that could affect outcomes (e.g., demographics, risk factors, co-morbid conditions)?  7. Was the overall drop-out rate from the study at endpoint 20% or lower of the number allocated to treatment?  8. Was the differential drop-out rate (between treatment groups) at endpoint 15 percentage points or lower?  9. Was there high adherence to the intervention protocols for each treatment group?  10. Were other interventions avoided or similar in the groups (e.g., similar background treatments)?  11. Were outcomes assessed using valid and reliable measures, implemented consistently across all study participants?  12. Did the authors report that the sample size was sufficiently large to be able to detect a difference in the main outcome between groups with at least 80% power?  13. Were outcomes reported or subgroups analyzed prespecified (i.e., identified before analyses were conducted)?  14. Were all randomized participants analyzed in the group to which they were originally assigned, i.e., did they use an intention-to-treat analysis?  **Criteria used to assess risk of bias of pre-post study without control group:**  1. Was the study question or objective clearly stated?  2. Were eligibility/selection criteria for the study population prespecified and clearly described?  3. Were the participants in the study representative of those who would be eligible for the test/service/intervention in the general or clinical population of interest?  4. Were all eligible participants that met the prespecified entry criteria enrolled?  5. Was the sample size sufficiently large to provide confidence in the findings?  6. Was the test/service/intervention clearly described and delivered consistently across the study population?  7. Were the outcome measures prespecified, clearly defined, valid, reliable, and assessed consistently across all study participants?  8. Were the people assessing the outcomes blinded to the participants' exposures/interventions?  9. Was the loss to follow-up after baseline 20% or less? Were those lost to follow-up accounted for in the analysis?  10. Did the statistical methods examine changes in outcome measures from before to after the intervention? Were statistical tests done that provided p values for the pre-to-post changes?  11. Were outcome measures of interest taken multiple times before the intervention and multiple times after the intervention (i.e., did they use an interrupted time-series design)?  12. If the intervention was conducted at a group level (e.g., a whole hospital, a community, etc.) did the statistical analysis take into account the use of individual-level data to determine effects at the group level? | | | | | | | | | | | | | | | | |  |

# Supplemental Table 5. Quality Assessment Tool for observational studies

| **Lead Author, Publication Year** | **Study Design** | **1** | **2** | **3** | **4** | **5** | **6** | **7** | **8** | **9** | **10** | **11** | **12** | **13** | **14** | **Methodological quality (Total score)^1^** | **Study level** |
| --- | --- | --- | --- | --- | --- | --- | --- | --- | --- | --- | --- | --- | --- | --- | --- | --- | --- |
| **Abilmona et al., 2018^35^** | Cross-sectional | Y | Y | NR | Y | N | N | NA | N | Y | N | Y | NR | NA | Y | Low (3/13,46.15%) | 4 |
| **Allison et al., 2018^36^** | Post-intervention follow-up study | Y | Y | N | Y | N | N | NA | N | Y | N | Y | NR | N | N | Low (5/14,35.7%) | 4 |
| **Beal et al. 2017^37^** | Case-Control | Y | Y | N | Y | Y | Y | NR | NR | NA | Y | NR | Y | --- | --- | Moderate (7/12, 58.3%) | 3 |
| **de Groot et al., 2013^38^** | Longitudinal study | Y | Y | Y | Y | N | Y | Y | N | Y | N | Y | NR | N | N | Moderate (10/14, 71.43%) | 3 |
| **DiPiro et al, 2019^39^** | Longitudinal | Y | Y | NR | Y | N | Y | Y | N | Y | N | Y | NR | N | N | Moderate (7/14, 50%) | 3 |
| **Goldsmith et al., 2022^402^** | Cross-sectional | Y | Y | NR | Y | Y | N | N | N | Y | N | Y | NR | NA | N | Low (6/13, 46.15%) | 4 |
| **Gorgey et al., 2015^41^** | Cross-sectional | Y | Y | Y | Y | N | N | N | N | Y | N | Y | NR | NA | N | Low (6/13, 46.15%) | 4 |
| **Javidan et al., 2017^42^** | Cross-sectional | Y | Y | Y | Y | N | N | N | N | Y | N | Y | NR | NA | N | Low (6/13, 46.15%) | 4 |
| **Li et al., 2021^43^** | Cross-sectional | Y | Y | Y | Y | N | N | N | N | Y | N | Y | NR | NA | Y | Moderate (7/13, 53.85%) | 4 |
| **Lieberman et al., 2014^44^** | Case-control | Y | Y | NR | Y | Y | N | N | Y | Y | N | Y | NR | NA | N | Moderate (7/13, 53.85%) | 3 |
| **Mohammadi et al., 2021^45^** | Cross-sectional | Y | Y | Y | Y | N | N | N | N | Y | N | Y | NR | NA | Y | Moderate (7/13, 53.85%) | 4 |
| Abbreviations: CD, cannot determine; NA, not applicable; NR, not reported  **^1^**Methodological quality rating (High (75-100%), Moderate (50-75%), or Low (<50%)  ^2^baseline analysis of two clinical trials  **Criteria used to asses risk of bias of cohort/cross-sectional studies:**  1. Was the research question or objective in this paper clearly stated?  2. Was the study population clearly specified and defined?  3. Was the participation rate of eligible persons at least 50%?  4. Were all the subjects selected or recruited from the same or similar populations (including the same time period)? Were inclusion and exclusion criteria for being in the study prespecified and applied uniformly to all participants?  5. Was a sample size justification, power description, or variance and effect estimates provided?  6. For the analyses in this paper, were the exposure(s) of interest measured prior to the outcome(s) being measured?  7. Was the timeframe sufficient so that one could reasonably expect to see an association between exposure and outcome if it existed?  8. For exposures that can vary in amount or level, did the study examine different levels of the exposure as related to the outcome (e.g., categories of exposure, or exposure measured as continuous variable)?  9. Were the exposure measures (independent variables) clearly defined, valid, reliable, and implemented consistently across all study participants?  10. Was the exposure(s) assessed more than once over time?  11. Were the outcome measures (dependent variables) clearly defined, valid, reliable, and implemented consistently across all study participants?  12. Were the outcome assessors blinded to the exposure status of participants?  13. Was loss to follow-up after baseline 20% or less?  14. Were key potential confounding variables measured and adjusted statistically for their impact on the relationship between exposure(s) and outcome(s)?  **Criteria used to asses risk of bias of case-control studies:**  1. Was the research question or objective in this paper clearly stated and appropriate?  2. Was the study population clearly specified and defined?  3. Did the authors include a sample size justification?  4. Were controls selected or recruited from the same or similar population that gave rise to the cases (including the same timeframe)?  5. Were the definitions, inclusion and exclusion criteria, algorithms or processes used to identify or select cases and controls valid, reliable, and implemented consistently across all study participants?  6. Were the cases clearly defined and differentiated from controls?  7. If less than 100 percent of eligible cases and/or controls were selected for the study, were the cases and/or controls randomly selected from those eligible?  8. Was there use of concurrent controls?  9. Were the investigators able to confirm that the exposure/risk occurred prior to the development of the condition or event that defined a participant as a case?  10. Were the measures of exposure/risk clearly defined, valid, reliable, and implemented consistently (including the same time period) across all study participants?  11. Were the assessors of exposure/risk blinded to the case or control status of participants?  12. Were key potential confounding variables measured and adjusted statistically in the analyses? If matching was used, did the investigators account for matching during study analysis? | | | | | | | | | | | | | | | | |  |

**References**

1. Li J, Polston KFL, Eraslan M, et al. A high-protein diet or combination exercise training to improve metabolic health in individuals with long-standing spinal cord injury: a pilot randomized study. *Physiol Rep* 2018;6(16):e13813. doi: 10.14814/phy2.13813 [published Online First: 2018/08/30]

2. Yarar-Fisher C, Kulkarni A, Li J, et al. Evaluation of a ketogenic diet for improvement of neurological recovery in individuals with acute spinal cord injury: a pilot, randomized safety and feasibility trial. *Spinal Cord Ser Cases* 2018;4:88. doi: <https://dx.doi.org/10.1038/s41394-018-0121-4>

3. Gorgey AS, Mather KJ, Cupp HR, et al. Effects of resistance training on adiposity and metabolism after spinal cord injury. *Med Sci Sports Exerc* 2012;44(1):165-74. doi: 10.1249/MSS.0b013e31822672aa [published Online First: 2011/06/11]

4. Myers J, Gopalan R, Shahoumian T, et al. Effects of customized risk reduction program on cardiovascular risk in males with spinal cord injury. *J Rehabil Res Dev* 2012;49(9):1355-64. doi: 10.1682/jrrd.2011.11.0215 [published Online First: 2013/02/15]

5. Mohammadi V, Khalili M, Eghtesadi S, et al. The effect of alpha-lipoic acid (ALA) supplementation on cardiovascular risk factors in men with chronic spinal cord injury: a clinical trial. *Spinal Cord* 2015;53(8):621-4. doi: 10.1038/sc.2015.35 [published Online First: 2015/03/11]

6. Zheng J, Liu J, Jiang Y, et al. Safety of Every-Other-Day Fasting in the Treatment of Spinal Cord Injury: A Randomized Controlled Trial. *American Journal of Physical Medicine & Rehabilitation* 2021;100(12):1184-89. doi: <https://dx.doi.org/10.1097/PHM.0000000000001727>

7. Radomski M, Finkelstein M, Hagel S, et al. A Pilot Wellness and Weight Management Program for Individuals with Spinal Cord Injury: Participants’ Goals and Outcomes. *Topics in Spinal Cord Injury Rehabilitation* 2011;17:59-69. doi: 10.1310/sci1702-59

8. Dolbow DR, Credeur DP, Lemacks JL, et al. Electrically induced cycling and nutritional counseling for counteracting obesity after spinal cord injury: A pilot study. *J Spinal Cord Med* 2021;44(4):533-40. doi: <https://dx.doi.org/10.1080/10790268.2019.1710939>

9. Javierre C, Vidal J, Segura R, et al. Continual supplementation with n-3 fatty acids does not modify plasma lipid profile in spinal cord injury patients. *Spinal Cord* 2005;43(9):527-30.

10. Szlachcic Y, Adkins RH, Adal T, et al. The effect of dietary intervention on lipid profiles in individuals with spinal cord injury. *J Spinal Cord Med* 2001;24(1):26-9. doi: 10.1080/10790268.2001.11753551 [published Online First: 2001/10/06]

11. Sabour H, Javidan AN, Soltani Z, et al. The effect of behavioral intervention and nutrition education program on serum lipid profile, body weight and blood pressure in Iranian individuals with spinal cord injury: A randomized clinical trial. *J Spinal Cord Med* 2018;41(1):28-35. doi: 10.1080/10790268.2016.1209890 [published Online First: 2016/08/26]

12. Javierre C, Vidal J, Segura R, et al. The effect of supplementation with n-3 fatty acids on the physical performance in subjects with spinal cord injury. *J Physiol Biochem* 2006;62(4):271-9.

13. Chen Y, Henson S, Jackson AB, et al. Obesity intervention in persons with spinal cord injury. *Spinal Cord* 2006;44(2):82-91.

14. Amorim S, Teixeira VH, Corredeira R, et al. Creatine or vitamin D supplementation in individuals with a spinal cord injury undergoing resistance training: A double-blinded, randomized pilot trial. *J Spinal Cord Med* 2018;41(4):471-78. doi: 10.1080/10790268.2017.1372058

15. Allison DJ, Josse AR, Gabriel DA, et al. Targeting inflammation to influence cognitive function following spinal cord injury: a randomized clinical trial. *Spinal Cord* 2017;55(1):26-32. doi: <https://dx.doi.org/10.1038/sc.2016.96>

16. Sabour H, Larijani B, Vafa MR, et al. The effects of n-3 fatty acids on inflammatory cytokines in osteoporotic spinal cord injured patients: A randomized clinical trial. *J Res Med Sci* 2012;17(4):322-7. [published Online First: 2012/12/26]

17. Jacobs PL, Mahoney ET, Cohn KA, et al. Oral creatine supplementation enhances upper extremity work capacity in persons with cervical-level spinal cord injury. *Archives of Physical Medicine & Rehabilitation* 2002;83(1):19-23.

18. Pritchett K, Pritchett RC, Stark L, et al. Effect of Vitamin D Supplementation on 25(OH)D Status in Elite Athletes With Spinal Cord Injury. *Int J Sport Nutr Exerc Metab* 2019;29(1):18-23. doi: <https://dx.doi.org/10.1123/ijsnem.2017-0233>

19. Kendall RW, Jacquemin G, Frost R, et al. Creatine supplementation for weak muscles in persons with chronic tetraplegia: a randomized double-blind placebo-controlled crossover trial. *J Spinal Cord Med* 2005;28(3):208-13.

20. Bauman WA, Spungen AM, Morrison N, et al. Effect of a vitamin D analog on leg bone mineral density in patients with chronic spinal cord injury. *J Rehabil Res Dev* 2005;42(5):625-34.

21. Aminmansour B, Asnaashari A, Rezvani M, et al. Effects of progesterone and vitamin D on outcome of patients with acute traumatic spinal cord injury; a randomized, double-blind, placebo controlled study. *J Spinal Cord Med* 2016;39(3):272-80. doi: <https://dx.doi.org/10.1080/10790268.2015.1114224>

22. Norouzi Javidan A, Sabour H, Latifi S, et al. Does consumption of polyunsaturated fatty acids influence on neurorehabilitation in traumatic spinal cord-injured individuals? A double-blinded clinical trial. *Spinal Cord* 2014;52(5):378-82. doi: 10.1038/sc.2014.30 [published Online First: 2014/03/19]

23. Allison DJ, Gabriel DA, Klentrou P, et al. The Influence of Chronic Inflammation on Peripheral Motor Nerve Conduction Following Spinal Cord Injury: A Randomized Clinical Trial. *Topics in Spinal Cord Injury Rehabilitation* 2017;23(4):377-85. doi: <https://dx.doi.org/10.1310/sci16-00045>

24. Brewer S, Desneves K, Pearce L, et al. Effect of an arginine-containing nutritional supplement on pressure ulcer healing in community spinal patients. *J Wound Care* 2010;19(7):311-6.

25. Reid G, Hsiehl J, Potter P, et al. Cranberry juice consumption may reduce biofilms on uroepithelial cells: pilot study in spinal cord injured patients. *Spinal Cord* 2001;39(1):26-30.

26. Sappal S, Goetz LL, Vince R, et al. Randomized trial of concentrated proanthocyanidins (PAC) for acute reduction of bacteriuria in male veterans with spinal cord injury utilizing clean intermittent catheterization. *Spinal Cord Ser Cases* 2018;4:58. doi: 10.1038/s41394-018-0087-2 [published Online First: 2018/07/07]

27. Linsenmeyer TA, Harrison B, Oakley A, et al. Evaluation of cranberry supplement for reduction of urinary tract infections in individuals with neurogenic bladders secondary to spinal cord injury. A prospective, double-blinded, placebo-controlled, crossover study. *J Spinal Cord Med* 2004;27(1):29-34. doi: 10.1080/10790268.2004.11753727

28. Waites KB, Canupp KC, Armstrong S, et al. Effect of cranberry extract on bacteriuria and pyuria in persons with neurogenic bladder secondary to spinal cord injury. *J Spinal Cord Med* 2004;27(1):35-40.

29. Hess MJ, Hess PE, Sullivan MR, et al. Evaluation of cranberry tablets for the prevention of urinary tract infections in spinal cord injured patients with neurogenic bladder. *Spinal Cord* 2008;46(9):622-6. doi: 10.1038/sc.2008.25 [published Online First: 2008/04/09]

30. Toh SL, Lee BB, Ryan S, et al. Probiotics [LGG-BB12 or RC14-GR1] versus placebo as prophylaxis for urinary tract infection in persons with spinal cord injury [ProSCIUTTU]: a randomised controlled trial. *Spinal Cord* 2019;57(7):550-61. doi: <https://dx.doi.org/10.1038/s41393-019-0251-y>

31. Lee BB, Haran MJ, Hunt LM, et al. Spinal-injured neuropathic bladder antisepsis (SINBA) trial. *Spinal Cord* 2007;45(8):542-50. doi: 10.1038/sj.sc.3101974 [published Online First: 2006/10/18]

32. Li J, Morrow C, McLain A, et al. Effects of a Low-Carbohydrate, High-Protein Diet on Gut Microbiome Composition in Insulin-Resistant Individuals With Chronic Spinal Cord Injury: Preliminary Results From a Randomized Controlled Trial. *Archives of Physical Medicine & Rehabilitation* 2022;103(7):1269-78. doi: <https://dx.doi.org/10.1016/j.apmr.2022.03.014>

33. Wong S, Jamous A, O'Driscoll J, et al. A Lactobacillus casei Shirota probiotic drink reduces antibiotic-associated diarrhoea in patients with spinal cord injuries: a randomised controlled trial. *Br J Nutr* 2014;111(4):672-8. doi: 10.1017/S0007114513002973 [published Online First: 2013/09/21]

34. Allison DJ, Ditor DS. Targeting inflammation to influence mood following spinal cord injury: a randomized clinical trial. *J Neuroinflammation* 2015;12:204. doi: <https://dx.doi.org/10.1186/s12974-015-0425-2>

35. Abilmona SM, Gorgey AS. Associations of the trunk skeletal musculature and dietary intake to biomarkers of cardiometabolic health after spinal cord injury. *Clin Physiol Funct Imaging* 2018;06:06. doi: <https://dx.doi.org/10.1111/cpf.12505>

36. Allison DJ, Ditor DS. Maintenance of diet participation in individuals with spinal cord injury: effect on mood and neuropathic pain. *Spinal Cord Ser Cases* 2018;4:97. doi: <https://dx.doi.org/10.1038/s41394-018-0131-2>

37. Beal C, Gorgey A, Moore P, et al. Higher dietary intake of vitamin D may influence total cholesterol and carbohydrate profile independent of body composition in men with Chronic Spinal Cord Injury. *J Spinal Cord Med* 2018;41(4):459-70. doi: <https://dx.doi.org/10.1080/10790268.2017.1361561>

38. de Groot S, Post MW, Snoek GJ, et al. Longitudinal association between lifestyle and coronary heart disease risk factors among individuals with spinal cord injury. *Spinal Cord* 2013;51(4):314-8. doi: <https://dx.doi.org/10.1038/sc.2012.153>

39. DiPiro ND, Cao Y, Krause JS. A prospective study of health behaviors and risk of all-cause and cause-specific mortality after spinal cord injury. *Spinal Cord* 2019;57(11):933-41. doi: <https://dx.doi.org/10.1038/s41393-019-0298-9>

40. Goldsmith JA, Holman ME, Puri P, et al. The interaction of macronutrients and body composition among individuals with chronic spinal cord injury. *Br J Nutr* 2022:1-32. doi: <https://dx.doi.org/10.1017/S0007114522001830>

41. Gorgey AS, Caudill C, Sistrun S, et al. Frequency of Dietary Recalls, Nutritional Assessment, and Body Composition Assessment in Men With Chronic Spinal Cord Injury. *Archives of Physical Medicine & Rehabilitation* 2015;96(9):1646-53. doi: <https://dx.doi.org/10.1016/j.apmr.2015.05.013>

42. Javidan AN, Sabour H, Nazari M, et al. Is the pattern of dietary amino acids intake associated with serum lipid profile and blood pressure among individuals with spinal cord injury? *J Spinal Cord Med* 2017;40(2):201-12. doi: <https://dx.doi.org/10.1080/10790268.2015.1109761>

43. Li J, Demirel A, Azuero A, et al. Limited Association between the Total Healthy Eating Index-2015 Score and Cardiovascular Risk Factors in Individuals with Long-Standing Spinal Cord Injury: An Exploratory Study: An Exploratory Study. *J Acad Nutr Diet* 2021;17:17. doi: <https://dx.doi.org/10.1016/j.jand.2021.04.010>

44. Lieberman J, Goff D, Jr., Hammond F, et al. Dietary intake relative to cardiovascular disease risk factors in individuals with chronic spinal cord injury: a pilot study. *Topics in Spinal Cord Injury Rehabilitation* 2014;20(2):127-36. doi: <https://dx.doi.org/10.1310/sci2002-127>

45. Mohammadi H, Parastouei K, Rostami H, et al. The association between dietary inflammatory index and psychological profile among men with spinal cord injury. *J Spinal Cord Med* 2021:1-6. doi: <https://dx.doi.org/10.1080/10790268.2021.1905977>

Appendix I

Detailed search strategies used in this review

**The role of diet (and nutritional supplementation) on health outcomes in spinal cord injured population: a systematic review.
July 11, 2022 (date last searched)**

|  | Before deduplication | After deduplication |
| --- | --- | --- |
| Embase.com | 7947 |  |
| MEDLINE (Ovid) | 5684 |  |
| Cochrane Library | 559 (22 Cochrane Reviews; 537 Trials) |  |
| Web of Science | 4544 |  |
| Google Scholar | 200 |  |
| **Total** | **18'934** | **12'313** |

6'621 duplicate records have been removed

**Embase.com**

('spinal cord injury'/exp OR 'cervical spine injury'/de OR 'spinal cord ischemia'/de OR 'paraplegia'/de OR 'spastic paraplegia'/de OR 'quadriplegia'/de OR 'spinal dysraphism'/de OR 'spinal cord contusion'/de OR 'spinal cord transsection'/de OR 'spinal paralysis'/de OR ('injury'/exp AND 'spinal cord'/exp) OR 'disabled athlete'/exp OR 'wheelchair athlete'/de OR (((spine or spinal) NEAR/3 (injur* or trauma* or damag* OR rupture* OR paralysis)) OR (spinal cord NEAR/3 (disease* or contusion* or laceration* or transection* or lesion* or trauma* or ischemi* or ischaemi*)) OR (myelopath* NEAR/3 (trauma* or post-trauma* or posttrauma*)) OR ((spine or spinal or vertebrae) NEAR/3 (fracture* or trauma* or injur* or damage* or wound* or impairment*)) OR 'central cord injury syndrome*' OR 'central cord syndrome*' OR 'central spinal cord syndrome*' OR 'cauda equine syndrome*' OR 'anterior cord syndrome*' OR 'conus medullaris syndrome*' OR 'Brown Sequard' OR paraplegi* OR quadriplegi* OR tetraplegi* OR 'spina bifida' OR paralympi* OR para-athlet* OR parathlet* OR para-sport* OR parasport* OR (athlet* NEAR/3 disabilit*)):ti,ab,kw) **AND** ('diet'/exp OR 'diet therapy'/exp OR 'diet supplementation'/de OR 'dietary intake'/exp OR 'eating'/de OR 'nutrition'/exp OR 'sport nutrition'/de OR 'food'/exp OR 'food intake'/exp OR 'nutrient'/exp OR 'caloric intake'/de OR 'caloric restriction'/de OR 'feeding behavior'/exp OR 'protein intake'/de OR 'carbohydrate intake'/exp OR 'carbohydrate diet'/exp OR 'fiber intake'/de OR 'dietary fiber'/exp OR 'plant protein'/exp OR 'vegetable'/exp OR 'fat intake'/exp OR 'fatty acid'/exp OR 'dietary supplement'/de OR 'probiotic agent'/exp OR (diet OR diets OR dietary OR dieti* OR nutri* OR macronutrient* OR macro-nutrient* OR micronutrient* OR micro-nutrient* OR food* OR eating OR meal OR meals OR fasting OR dairy OR milk OR probiotic* OR nutraceutic* OR lactobacill* OR bifidobacter* OR ((sugar* OR sweet* OR flavor* OR flavour*) NEAR/3 (drink* OR beverage* OR soda*)) OR ((sugar OR carbohydrate* OR fat OR calori* OR energy OR protein* OR fiber* OR fibre*) NEAR/2 (intake OR consumption* OR consumed OR feeding)) OR (protein NEXT/1 supplement*) OR 'fatty acid*' OR 'saturated fat*' OR 'monounsaturated fat*' OR 'mono-unsaturated fat*' OR 'polyunsaturated fat*' OR 'poly-unsaturated fat*' OR 'trans fat*' OR 'fish oil*' OR 'olive oil*' OR omega-3 OR omega-6 OR 'linolenic acid*' OR 'linoleic acid*' OR alcohol* OR coffee OR caffeine OR tea OR beer OR wine OR juic* OR eggs OR fruit* OR meat OR meats OR poultry OR fish OR seafood OR nuts OR seeds OR 'whole grain*' OR vegetable* OR legume* OR ((diet* OR nutri*) NEAR/3 (fat* OR protein*)) OR 'plant protein*' OR carbohydrate* OR sugar* OR sweets OR pastries OR confectionery OR candy OR sodium OR nitrate* OR creatine OR leucine OR beta-alanine* OR 'pantothenic acid*' OR vitamin* OR zinc OR magnesium OR 'trace element*'):ab,ti,kw) NOT ([animals]/lim NOT [humans]/lim) NOT ([Conference Abstract]/lim OR [Letter]/lim OR [Note]/lim OR [Editorial]/lim OR [preprint]/lim) NOT 'case-report*':ti

**MEDLINE (Ovid)**

(exp Spinal Cord Injuries/ OR exp Spinal Cord Ischemia/ OR exp Paraplegia/ OR Quadriplegia/ OR Spinal Dysraphism/ OR exp Spinal Injuries/ OR ("Wounds and Injuries"/ AND Spinal Cord/) OR Para-Athletes/ OR (((spine or spinal) adj3 (injur* or trauma* or damag* OR rupture* OR paralysis)) OR (spinal cord adj3 (disease* or contusion* or laceration* or transection* or lesion* or trauma* or ischemi* or ischaemi*)) OR (myelopath* adj3 (trauma* or post-trauma* or posttrauma*)) OR ((spine or spinal or vertebrae) adj3 (fracture* or trauma* or injur* or damage* or wound* or impairment*)) OR 'central cord injury syndrome*' OR 'central cord syndrome*' OR 'central spinal cord syndrome*' OR 'cauda equine syndrome*' OR 'anterior cord syndrome*' OR 'conus medullaris syndrome*' OR 'Brown Sequard' OR paraplegi* OR quadriplegi* OR tetraplegi* OR 'spina bifida' OR paralympi* OR para-athlet* OR parathlet* OR para-sport* OR parasport* OR (athlet* adj3 disabilit*)).ti,ab,kf.) AND (exp Diet/ OR exp Diet Therapy/ OR exp Food/ OR exp Eating/ OR Sports Nutritional Physiological Phenomena/ OR Nutritional Status/ OR exp Feeding Behavior/ OR exp Dietary Proteins/ OR exp Dietary Carbohydrates/ OR exp Dietary Fiber/ OR exp Plant Proteins/ OR exp Dietary Fats/ OR exp Fatty Acids/ OR (diet OR diets OR dietary OR dieti* OR nutri* OR macronutrient* OR macro-nutrient* OR micronutrient* OR micro-nutrient* OR food* OR eating OR meal OR meals OR fasting OR dairy OR milk OR probiotic* OR nutraceutic* OR lactobacill* OR bifidobacter* OR ((sugar* OR sweet* OR flavor* OR flavour*) adj3 (drink* OR beverage* OR soda*)) OR ((sugar OR carbohydrate* OR fat OR calori* OR energy OR protein* OR fiber* OR fibre*) adj2 (intake OR consumption* OR consumed OR feeding)) OR (protein adj supplement*) OR fatty acid* OR saturated fat* OR monounsaturated fat* OR mono-unsaturated fat* OR polyunsaturated fat* OR poly-unsaturated fat* OR trans fat* OR fish oil* OR olive oil* OR omega-3 OR omega-6 OR linolenic acid* OR linoleic acid* OR alcohol* OR coffee OR caffeine OR tea OR beer OR wine OR juic* OR eggs OR fruit* OR meat OR meats OR poultry OR fish OR seafood OR nuts OR seeds OR whole grain* OR vegetable* OR legume* OR ((diet* OR nutri*) adj3 (fat* OR protein*)) OR plant protein* OR carbohydrate* OR sugar* OR sweets OR pastries OR confectionery OR candy OR sodium OR nitrate* OR creatine OR leucine OR beta-alanine* OR "pantothenic acid*" OR vitamin* OR zinc OR magnesium OR "trace element*").ab,ti,kf.) NOT (exp animals/ NOT humans/) NOT (letter OR news OR comment OR editorial OR congress).pt. NOT case-report*.ti

**Cochrane Library**

((((spine or spinal) NEAR/3 (injur* or trauma* or damag* OR rupture* OR paralysis)) OR (spinal-cord NEAR/3 (disease* or contusion* or laceration* or transection* or lesion* or trauma* or ischemi* or ischaemi*)) OR (myelopath* NEAR/3 (trauma* or post-trauma* or posttrauma*)) OR ((spine or spinal or vertebrae) NEAR/3 (fracture* or trauma* or injur* or damage* or wound* or impairment*)) OR central-cord-injury-syndrome* OR central-cord-syndrome* OR central-spinal-cord-syndrome* OR cauda-equine-syndrome* OR anterior-cord-syndrome* OR conus-medullaris-syndrome* OR "Brown Sequard" OR paraplegi* OR quadriplegi* OR tetraplegi* OR "spina bifida" OR paralympi* OR para-athlet* OR parathlet* OR para-sport* OR parasport* OR (athlet* NEAR/3 disabilit*)):ti,ab) AND ((diet OR diets OR dietary OR dieti* OR nutri* OR macronutrient* OR macro-nutrient* OR micronutrient* OR micro-nutrient* OR food* OR eating OR meal OR meals OR fasting OR dairy OR milk OR probiotic* OR nutraceutic* OR lactobacill* OR bifidobacter* OR ((sugar* OR sweet* OR flavor* OR flavour*) NEAR/3 (drink* OR beverage* OR soda*)) OR ((sugar OR carbohydrate* OR fat OR calori* OR energy OR protein* OR fiber* OR fibre*) NEAR/2 (intake OR consumption* OR consumed OR feeding)) OR protein-supplement* OR fatty-acid* OR saturated-fat* OR monounsaturated-fat* OR mono-unsaturated-fat* OR polyunsaturated-fat* OR poly-unsaturated-fat* OR trans-fat* OR fish-oil* OR omega-3 OR omega-6 OR linolenic-acid* OR linoleic-acid* OR alcohol* OR coffee OR caffeine OR tea OR beer OR wine OR juic* OR eggs OR fruit* OR meat OR meats OR seafood OR nuts OR seeds OR whole-grain* OR vegetable* OR legume* OR ((diet* OR nutri*) NEAR/3 (fat* OR protein*)) OR plant-protein* OR carbohydrate* OR sugar* OR sweets OR pastries OR confectionery OR candy OR sodium OR nitrate* OR creatine OR leucine OR beta-alanine* OR pantothenic-acid* OR vitamin* OR zinc OR magnesium OR trace-element*):ab,ti)

**Web of Science Core Collection**

TS=(((((spine or spinal) NEAR/2 (injur* or trauma* or damag* OR rupture* OR paralysis)) OR ("spinal cord" NEAR/2 (disease* or contusion* or laceration* or transection* or lesion* or trauma* or ischemi* or ischaemi*)) OR (myelopath* NEAR/2 (trauma* or post-trauma* or posttrauma*)) OR ((spine or spinal or vertebrae) NEAR/2 (fracture* or trauma* or injur* or damage* or wound* or impairment*)) OR "central cord injury syndrome*" OR "central cord syndrome*" OR "central spinal cord syndrome*" OR "cauda equine syndrome*" OR "anterior cord syndrome*" OR "conus medullaris syndrome*" OR "Brown Sequard" OR paraplegi* OR quadriplegi* OR tetraplegi* OR "spina bifida" OR paralympi* OR para-athlet* OR parathlet* OR para-sport* OR parasport* OR (athlet* NEAR/2 disabilit*))) AND ((diet OR diets OR dietary OR dieti* OR nutri* OR macronutrient* OR macro-nutrient* OR micronutrient* OR micro-nutrient* OR food* OR eating OR meal OR meals OR fasting OR dairy OR milk OR probiotic* OR nutraceutic* OR lactobacill* OR bifidobacter* OR ((sugar* OR sweet* OR flavor* OR flavour*) NEAR/2 (drink* OR beverage* OR soda*)) OR ((sugar OR carbohydrate* OR fat OR calori* OR energy OR protein* OR fiber* OR fibre*) NEAR/2 (intake OR consumption* OR consumed OR feeding)) OR "protein supplement*" OR "fatty acid*" OR "saturated fat*" OR "monounsaturated fat*" OR mono-unsaturated-fat* OR "polyunsaturated fat*" OR poly-unsaturated-fat* OR "trans fat*" OR "fish oil*" OR omega-3 OR omega-6 OR "linolenic acid*" OR "linoleic acid*" OR alcohol* OR coffee OR caffeine OR tea OR beer OR wine OR juic* OR eggs OR fruit* OR meat OR meats OR seafood OR nuts OR seeds OR "whole grain*" OR vegetable* OR legume* OR ((diet* OR nutri*) NEAR/2 (fat* OR protein*)) OR "plant protein*" OR carbohydrate* OR sugar* OR sweets OR pastries OR confectionery OR candy OR sodium OR nitrate* OR creatine OR leucine OR beta-alanine* OR pantothenic-acid* OR vitamin* OR zinc OR magnesium OR trace-element*))) NOT TS=(((animal* OR rat OR rats OR mouse OR mice OR rodent OR squirrel OR murine OR nonhuman* OR primate*) NOT (human* OR patient* OR women OR woman OR men OR man))) NOT TI=(case-report*) AND DT=(Article OR Early Access)

**Google Scholar** (first 200, sorted by relevance) of 1'240'000 results

"spinal cord" injury|injuries|trauma|damage|rupture|fracture|paralysis|paraplegia|tetraplegia|paralympic|"para athletes|athlete" diet|dietary|nutrition|nutritional|food|eating|fasting|macronutrient|micronutrient|"carbohydrate|energy|fat|protein intake"
